# In-depth metaproteomics analysis of tongue coating for gastric cancer: a multicenter diagnostic research study

**DOI:** 10.1186/s40168-023-01730-8

**Published:** 2024-01-08

**Authors:** Jiahui Chen, Yingying Sun, Jie Li, Mengge Lyu, Li Yuan, Jiancheng Sun, Shangqi Chen, Can Hu, Qing Wei, Zhiyuan Xu, Tiannan Guo, Xiangdong Cheng

**Affiliations:** 1 Department of Gastric Surgery, Zhejiang Cancer Hospital, Hangzhou Institute of Medicine (HIM), Chinese Academy of Sciences, Hangzhou, China; 2 Hangzhou Institute of Medicine (HIM), Chinese Academy of Sciences, Hangzhou, China; 3 Key Laboratory of Prevention, Diagnosis and Therapy of Upper Gastrointestinal Cancer of Zhejiang Province, Hangzhou, China; 4 Westlake Center for Intelligent Proteomics, Westlake Laboratory of Life Sciences and Biomedicine, Hangzhou, China; 5 School of Medicine, School of Life Sciences, Westlake University, Hangzhou, China; 6 Research Center for Industries of the Future, Westlake University, Hangzhou, China; 7 Department of Basic Medical Sciences, School of Medicine, Tsinghua University, Beijing, China; 8 MOE Key Laboratory of Bioinformatics, Tsinghua University, Beijing, China; 9 Department of Gastrointestinal Surgery, The Affiliated Hospital of Wenzhou Medical University, Wenzhou, China; 10 Department of General Surgery, HwaMei Hospital, University of Chinese Academy of Sciences, Ningbo, China

**Keywords:** Tongue coating, Metaproteomics, Gastric cancer, Noninvasive screening

## Abstract

**Background:**

Our previous study revealed marked differences in tongue images between individuals with gastric cancer and those without gastric cancer. However, the biological mechanism of tongue images as a disease indicator remains unclear. Tongue coating, a major factor in tongue appearance, is the visible layer on the tongue dorsum that provides a vital environment for oral microorganisms. While oral microorganisms are associated with gastric and intestinal diseases, the comprehensive function profiles of oral microbiota remain incompletely understood. Metaproteomics has unique strength in revealing functional profiles of microbiota that aid in comprehending the mechanism behind specific tongue coating formation and its role as an indicator of gastric cancer.

**Methods:**

We employed pressure cycling technology and data-independent acquisition (PCT-DIA) mass spectrometry to extract and identify tongue-coating proteins from 180 gastric cancer patients and 185 non-gastric cancer patients across 5 independent research centers in China. Additionally, we investigated the temporal stability of tongue-coating proteins based on a time-series cohort. Finally, we constructed a machine learning model using the stochastic gradient boosting algorithm to identify individuals at high risk of gastric cancer based on tongue-coating microbial proteins.

**Results:**

We measured 1432 human-derived proteins and 13,780 microbial proteins from 345 tongue-coating samples. The abundance of tongue-coating proteins exhibited high temporal stability within an individual. Notably, we observed the downregulation of human keratins KRT2 and KRT9 on the tongue surface, as well as the downregulation of ABC transporter COG1136 in microbiota, in gastric cancer patients. This suggests a decline in the defense capacity of the lingual mucosa. Finally, we established a machine learning model that employs 50 microbial proteins of tongue coating to identify individuals at a high risk of gastric cancer, achieving an area under the curve (AUC) of 0.91 in the independent validation cohort.

**Conclusions:**

We characterized the alterations in tongue-coating proteins among gastric cancer patients and constructed a gastric cancer screening model based on microbial-derived tongue-coating proteins. Tongue-coating proteins are shown as a promising indicator for identifying high-risk groups for gastric cancer.

Video Abstract

**Supplementary Information:**

The online version contains supplementary material available at 10.1186/s40168-023-01730-8.

## Background

Gastric cancer is one of the most common gastrointestinal malignancies in the world. According to statistics from the Global Cancer Observatory (GLOBOCAN), in 2020, gastric cancer accounted for over one million new cases and an estimated 769,000 deaths worldwide, making it the fifth most prevalent cancer and the fourth leading cause of cancer-related mortality [1]. Advanced gastric cancer’s prognosis remains unsatisfactory due to its high heterogeneity and aggressiveness. In contrast, early gastric cancer can achieve a favorable prognosis with standard treatment [2]. Endoscopy is one of the most reliable invasive screening methods for gastric cancer diagnosis [3, 4]. However, the widespread adoption of endoscopy is hindered by the need for specialized equipment and experienced medical professionals, as well as people’s reluctance due to the associated discomfort. Hence, there is an immediate requirement for a precise and widely applicable method to identify individuals at a high risk of developing gastric cancer.

The oral cavity is directly linked to the digestive tract, providing a valuable perspective for assessing the stomach’s condition. The tongue coating is a visible layer that adheres to the tongue dorsum and comprises deceased epithelial cells, blood metabolites, microorganisms, secretions from the postnasal area and the gingiva, and saliva [5]. Traditional Chinese medicine has a rich history of using the colors and thickness variations in tongue coating as indicators of an individual’s health status, aiding in the prediction or diagnosis [6] of conditions such as gastritis [7], nonalcoholic fatty liver disease [8], and colorectal cancer [9]. Our previous study revealed notable distinctions in tongue images between individuals with gastric cancer and those without gastric cancer. Moreover, the AI-based (artificial intelligence-based) gastric cancer screening model, leveraging tongue image features, exhibited precise identification of high-risk groups for gastric cancer [10]. However, the mechanism behind the development of distinct tongue coating in gastric cancer patients remains incompletely understood.

Microorganisms play an important role in the formation of tongue coating [11, 12]. The oral cavity hosts a vast array of microorganisms and is regarded as the second most intricate microbial community in the human body [13]. In recent years, increasing attention has been given to the relationship between oral microorganisms and digestive system malignancies. *Porphyromonas gingivalis* and Actinobacteria are associated with a higher risk of pancreatic cancer [14]. The gingival pathogen *Streptomyces forsythiae* is associated with a higher risk of esophageal cancer [15]. The oral pathogens *Treponema intermedia* and *Prevotella intermedia* are associated with an increased risk of colorectal cancer [16]. To date, several studies have revealed the oral microbial composition and function of gastric cancer patients by metagenomic or 16S rRNA sequencing, exploring the relationship between the oral microbiome and gastric cancer at the genomic level [17–20]. However, the presence of genes does not guarantee protein expression. Proteomics offers a promising approach to uncovering the functional profiles of oral microorganisms and the host, as it enables the simultaneous measurement of both the human and microbial proteins that are actively expressed. This enables the deciphering of host–microbe interactions in complex oral ecosystems [21, 22]. At present, research on oral metaproteomics is still in its infancy, most of which focuses on dental caries or oral malignancies. Only one study revealed the relationship between oral metaproteomics and lung cancer [23], and there is no research on oral metaproteomics for gastric cancer patients. Analyzing tongue-coating proteins of gastric cancer patients is crucial for investigating the connection between oral microorganisms and the onset and progression of gastric cancer.

Here, we carried out diagnostic research by collecting tongue coating from 180 individuals with gastric cancer and 185 individuals without gastric cancer, all gathered from five independent research centers located in China. We measured 1432 human proteins and 13,780 microbial proteins in these tongue-coating samples based on PCT-DIA mass spectrometry (MS). We validated the temporal stability of microbial proteins and human tongue-coating proteins based on a time-series cohort. We also found similar functional and taxonomic profiles of tongue-coating microbiota across different cohorts. Moreover, we discovered that the *Aminipila* genus exhibited a higher risk of gastric cancer with a medium odds ratio (OR) of 1.38 and 5.07 in two independent cohorts, which has not been previously reported. Notably, we observed the downregulation of keratins KRT2 and KRT9 on the tongue surface, as well as the ABC transporter COG1136 in microbiota, in gastric cancer patients, suggesting a decrease in lingual mucosa defense ability. Finally, we established a machine learning model using 50 microbial proteins from tongue coating to classify gastric cancer patients and non-gastric cancer individuals, achieving an AUC of 0.91 in the independent validation cohort.

## Methods

### Tongue coating sample collection

The study stipulated that patients aged 18 to 80 years with histologically confirmed adenocarcinoma of the stomach or gastroesophageal junction were eligible. Any patients who had previously been treated for gastric cancer (including with medication, radiation, or surgery) or had oral diseases were excluded. The noncancer samples were donated by healthy volunteers with no history of cancer, negative screening for gastrointestinal tumors, and no oral diseases.

**Fig. 1:**
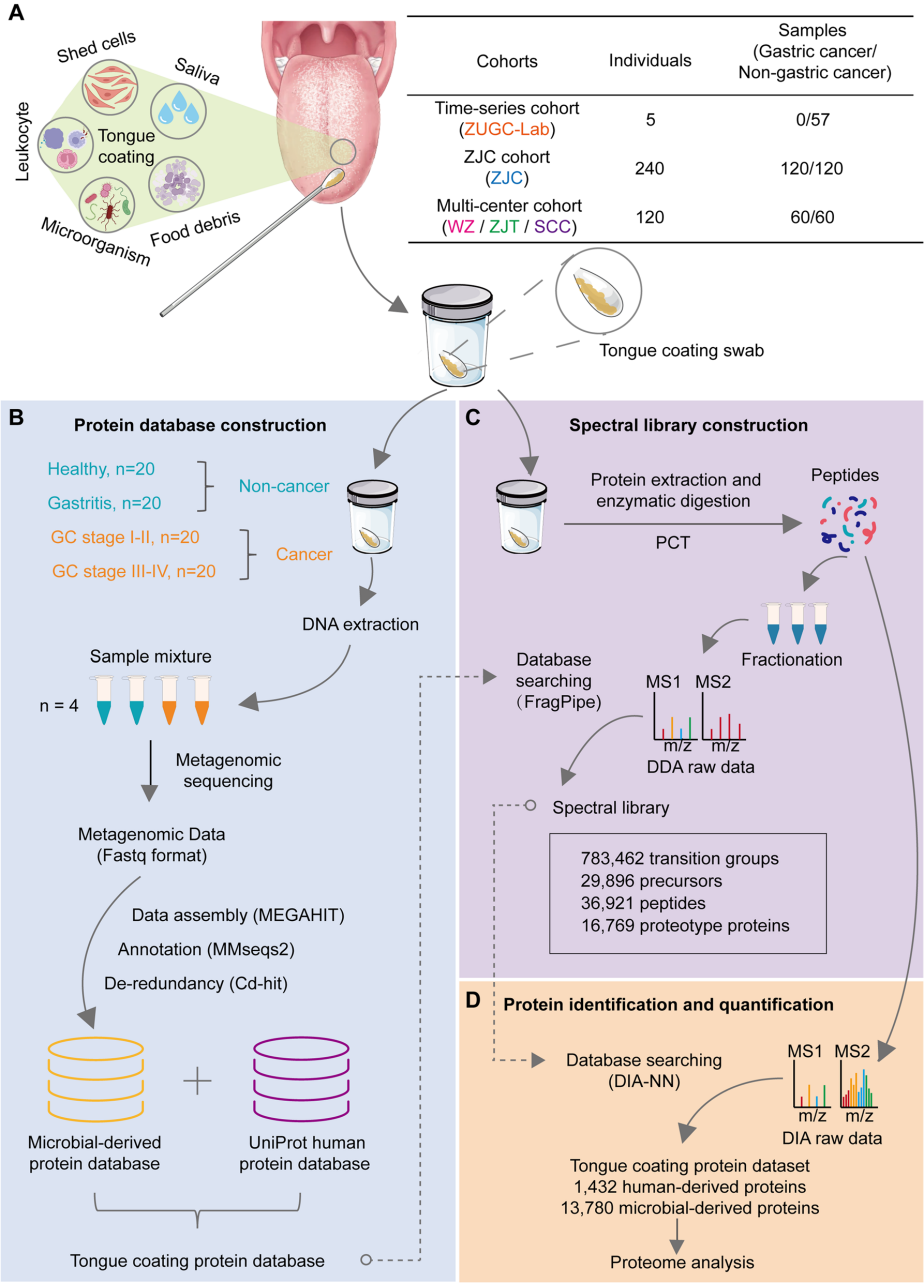
Schematic view of the study. **A** The main components of tongue coating and cohort information included in this study. **B** Process of constructing the tongue-coating protein database. **C** Construction of the spectral library of tongue-coating proteins. **D** Tongue-coating protein identification and quantification workflow based on PCT-DIA

The tongue coating of gastric cancer patients was collected on the morning of the operation day. The tongue coating of non-cancer volunteers was collected in the morning 1 week after gastroscopy. Disposable swabs were used to collect tongue-coating samples from participants before the consumption of breakfast or water. Before taking tongue-coating samples, participants used sterile water to rinse their mouths three times. The tongue was scraped from the root to the tip 15 times (each swab was rolled 5 times, for a total of 3 swabs) by simultaneously rolling the swab with a professional operator (Fig. 1A). Then, the swab was immediately placed into a storage tube and transferred to the freezer at − 80 °C.

### Construction of the tongue-coating protein database

Due to differences in regions, diets, and health conditions, the existing public protein databases cannot fully reflect the proteome characteristics of the tongue coating of gastric cancer patients. Therefore, we constructed a tongue-coating protein database through metagenomic sequencing to support DIA-MS protein identification and quantification. We randomly selected 20 tongue-coating samples from healthy individuals, patients with gastritis, patients with stage I–II tumors, and patients with stage III–IV tumors, respectively. DNA was extracted from each sample, and then, DNA samples from the same group were subsequently pooled for metagenomic sequencing. Consequently, metagenomic sequencing was carried out on four mixed DNA samples, each representing one of the four groups mentioned above (Fig. 1B, Supplemental Fig. 1A). The detailed protocol of DNA extraction and metagenomic sequencing is described as follows.

DNA from different tongue-coating samples was extracted using the E.Z.N.A.® Stool DNA Kit (D4015-02, Omega, Inc., USA) according to the manufacturer’s instructions. The reagent, specifically designed for the detection of DNA in trace sample amounts, has proven to be effective in extracting DNA from the majority of bacteria. Sample blanks included unused swabs that underwent the DNA extraction process and were confirmed to contain no DNA amplicons. The total DNA was eluted in 50 µl of elution buffer using a modification of the manufacturer’s procedure (QIAGEN) and then stored at − 80 °C.

The DNA library was constructed using the TruSeq Nano DNA LT Library Preparation Kit (FC-121–4001). DNA was fragmented by dsDNA Fragmentase (NEB, M0348S) and incubated at 37 °C for 30 min. Blunt-end DNA fragments were generated using a combination of fill-in reactions and exonuclease activity. Size selection was performed with the provided sample purification beads. An A-base was then added to the blunt ends of each strand, preparing them for ligation to the indexed adapters. Each adapter contains a T-base overhang for ligating the adapter to the A-tailed fragmented DNA. These adapters encompass the complete set of sequencing primer hybridization sites for single, paired-end, and indexed reads. Single- or dual-index adapters were ligated to the fragments, and the ligated products were amplified with PCR by the following conditions: initial denaturation at 95 °C for 3 min; 8 cycles of denaturation at 98 °C for 15 s, annealing at 60 °C for 15 s, and extension at 72 °C for 30 s; and a final extension at 72 °C for 5 min. Finally, we performed 2 × 150 bp paired-end sequencing (PE150) on an Illumina NovaSeq™ 6000 (LC-Bio Technology Co., Ltd., Hangzhou, China) following the vendor’s recommended protocol.

Raw sequencing reads were processed to obtain valid reads for further analysis. First, sequencing adapters were removed from sequencing reads using cutadapt v1.9. Then, low-quality reads were trimmed by fqtrim v0.94 using a sliding-window algorithm. The clean reads were assembled using MEGAHIT [24] (v1.2.9) with the parameters “min contig = 100, Kmer = 21, 33, 55, 77.” Cd-hit [25] (V4.8.1) was used for sequence redundancy (sequence identity threshold: 0.95, alignment coverage for the shorter sequence: 0.9). Finally, MMseqs2 [26] (v13.45111, default parameter) was used for protein annotation against the UniProt database (UniRef90). We combined the human protein data from the UniProt database (UP000005640) and the tongue-coating microbial database to construct the tongue-coating protein database (Fig. 1B, Supplemental Fig.1A).

### Sample preparation assisted by PCT

We constructed and optimized a tongue-coating protein extraction process for proteomic analysis. After snipping the tip of the swab containing the tongue coating, it was immersed in a lysis buffer. The protein was then lysed in a constant temperature metal bath set to 30℃ and 1200 rpm for 60 min. Subsequently, the proteins were precipitated with prechilled acetone and then transferred to a solution containing 30 μL lysis buffer (6 M urea, 2 M thiourea), 5 μL Tris (2-carboxyethyl) phosphine (TECP, 10 mM), and 2.5 μL iodoacetamide (IAA) (800 mM). In PCT-MicroTubes (Pressure Bioscience Inc., USA), samples were lysed, reduced, and hydroxylated at 30 °C using PCT (90 cycles, 45 000 psi, 25 s on-time, and 10 s off-time). Trypsin (enzyme-to-substrate ratio, 1:50; Hualishi Scientific, China) and LysC (enzyme-to-substrate ratio, 1:40; Hualishi Scientific, China) were then added, followed by PCT-assisted digestion (120 cycles, 20,000 psi, 50 s on-time, and 10 s off-time). Trifluoroacetic acid (TFA, 10%) was added to terminate the digestion process. The resulting peptides were desalted with 2% acetonitrile (ACN) and 0.1% TFA and reconstituted. Peptide concentrations were measured with a Nanoscan (Analytic Jena, Germany) at A280, and samples were stored at 4 °C for further analysis. Unless otherwise specified, all chemical reagents were obtained from Sigma‒Aldrich.

### DDA mass spectrometry acquisition for library generation

Peptides were first loaded onto the pre-column (5 mm*300 μm i.d.) at a pressure of 217.5 bar, then entered the analytical column (1.9 μm, 120 Å, 150 mm*75 μm i.d.) at a flow rate of 300 nL/min, and were analyzed using a 60-min liquid chromatography gradient (0 ~ 50 min, 5 ~ 27% mobile phase B phase; 50 ~ 60 min, 27 ~ 40% mobile phase B phase). The scanning parameters of mass spectrometry were PASEF MS and MS/MS (Q Exactive HF-X hybrid Quadrupole-Orbitrap, Thermo Fisher Scientific) mass scan range from 100 to 1700 m/z, 1/k0 scan range from 0.6 to 1.6, mobility peak detection threshold of 5000, PASEF MS/MS scan number of 10, charge range from 0 to 5, and peak detection threshold of 2500 cts/s.

### Spectral library construction

We used FragPipe (version 19.1, Nesvizhskii lab) and a two-step database search strategy to generate a spectral library specific to the tongue coating of gastric cancer individuals [27, 28]. In the first step, each DDA raw file was searched against the tongue-coating protein database with a false discovery rate (FDR) of 0.01. The simplified protein database for each raw file was generated by extracting the proteins in the PSM matrices. Then, all the simplified protein databases and human protein database (UniProt date: February 26, 2020) were merged as the combined database for the second step. In the second step, all the DDA raw files were searched against the combined database with an FDR of 0.01 to generate the final spectral library (Fig. 1C, Supplemental Fig. 1B). We show the characteristics of the spectral library in Supplement file 2 and Supplemental Fig. 1C-H.

### Quantitative analysis of tongue-coating samples by PulseDIA

The PulseDIA [29] acquisition of the sample was performed on a nanoflow DIONEX UltiMate 3000 RSLCnano System (Thermo Fisher Scientific, San Jose) coupled to a Q Exactive HF-X hybrid Quadrupole-Orbitrap (Thermo Fisher Scientific, San Jose). The number of MS injections is two. For each PulseDIA acquisition, 0.5 μg of peptides was injected and separated across a 30-min LC gradient (from 3 to 28% buffer B) at a flow rate of 300 nL/min (pre-column, 3 μm, 100 Å, 20 mm × 75 μm i.d.; analytical column, 1.9 μm, 120 Å, 150 mm × 75 μm i.d.). Buffer A was HPLC-grade water containing 2% ACN and 0.1% FA, while buffer B was ACN containing 2% H_2_O and 0.1% FA. The total time for re-equilibration and sample loading was approximately 15 min. The raw data acquired by PulseDIA was searched against the spectral library using DIA-NN to identify and quantify tongue-coating proteins [30] (Fig. 1D).

### Taxonomic annotation of microbial-derived peptides

Taxonomic annotation was performed using the peptide-centric taxonomic annotation software, Unipept [31] (version 3.0.2). Since missed cleavages cannot be directly matched with the Unipept database, we initiated an in silico digestion of the peptides using the “Advanced missed cleavage handling” rule in Unipept. Subsequently, we filtered out peptides with fewer than 5 or more than 50 amino acids before annotating them using Unipept, applying the “Equal I and L” rule. When dealing with peptides that corresponded to multiple filtered peptides and filtered peptides annotated to different taxa, we first determined if the taxa belonged to the same branch. If the filtered peptides were annotated to the same branch, we retained the narrowest taxa. Otherwise, we retained the broadest taxa.

### Functional annotation and enrichment of differentially expressed proteins

To explore the functions of tongue coating proteins, we used eggNOG-mapper [32] (version 2.0) for Clusters of Orthologous Groups of proteins (COG) annotation and GhostKOALA [33] (version 2.0) for KEGG orthologue (KO) annotation. Statistical analysis was performed using R software (version 4.2.0). Wilcoxon tests were performed for differential protein (DEP) analysis, with a threshold of *P* value less than 0.05 or BH-adjusted *P* value less than 0.05. Fisher’s exact test was used for the significance analysis of pathway enrichment, and a *P* value < 0.05 was considered statistically significant.

### Machine learning for gastric cancer classification

We randomly divided the training cohort into 75% training data and 25% internal validation data. A stochastic gradient boosting model (GBM) based on microbial proteins was constructed to classify individuals with gastric cancer and those without gastric cancer. The machine learning stochastic GBM algorithm was performed using the R package gbm (version 2.1.8.1) with the parameters “interaction.depth = 3, n.trees = 150, shrinkage = 0.1, n.minobsinnode = 10.” This approach estimated the prediction error by performing tenfold cross-validation in the training data. Subsequently, the top 50 features were used as inputs into the GBM to predict the performance of the features in classification in training data and internal validation data. Finally, the independent validation data were used to verify the robustness of the model.

## Results

### Study design and clinical characteristics

A total of 417 tongue-coating samples from 180 individuals with gastric cancer and 185 individuals without gastric cancer from five independent research centers were collected in this study (Fig. 1A). Fifty-seven samples of five individuals from the ZUGC-Lab (Key Laboratory of Prevention, Diagnosis, and Therapy of Upper Gastrointestinal Cancer of Zhejiang Province) constitute the time-series cohort. This cohort was used to investigate the stability of the tongue-coating proteome over time. The tongue-coating samples of 120 gastric cancer patients and 120 noncancer individuals were collected from Zhejiang Cancer Hospital (ZJC) to form the ZJC cohort. This cohort was used to explore the characteristics of tongue-coating proteins and construct the gastric cancer screening model. In addition, tongue-coating samples from 60 gastric cancer patients and 60 noncancer individuals from the First Affiliated Hospital of Wenzhou Medical University (WZ), Zhejiang Hospital of Traditional Chinese Medicine (ZJT), and Sichuan Cancer Hospital (SCC) constituted the Multi-center cohort, which was used to verify the protein characteristics found in the ZJC cohort and to validate the robustness of the screening model (Fig. 1A, Supplemental Fig. 2A). Across different cohorts, there were no significant differences in terms of sex, age, drinking history, smoking history, etc., between the gastric cancer group and the non-cancer group (Supplemental Table 1). Clinical information for all individuals involved in the study is shown in Supplemental File 1. Quality control of tongue coating proteomics is shown in Supplemental Fig. 2. We excluded samples with fewer than 800 identified human-derived proteins or fewer than 2000 identified microbial-derived proteins. Finally, 233 samples from the ZJC cohort and 112 samples from the multi-center cohort were included in the follow-up analysis.

### The stability of the tongue-coating proteome over time

**Fig. 2:**
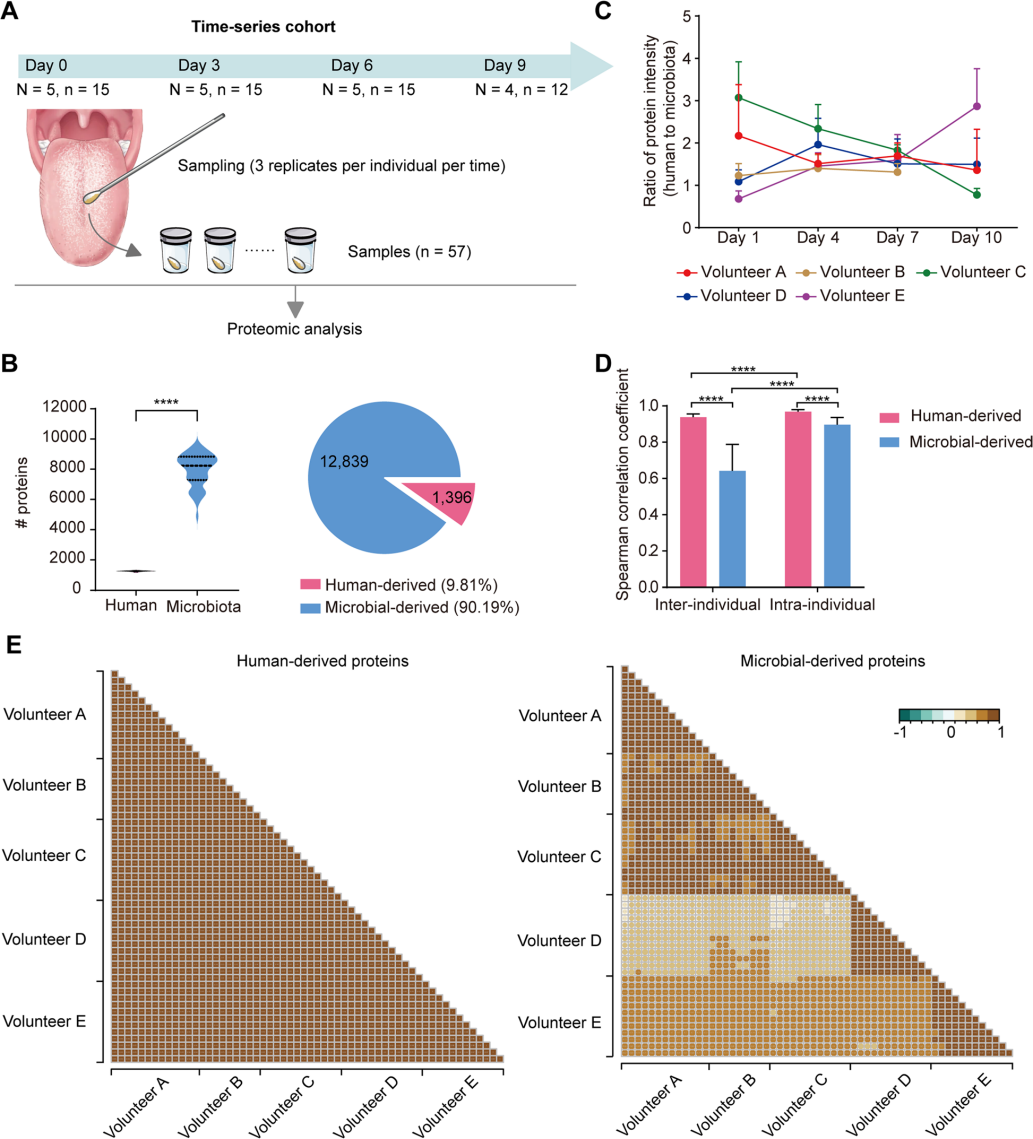
Stability of the tongue-coating proteome over time. **A** Collection and sample processing of the samples in the time-series cohort. **B** The number of human-derived proteins and microbial-derived proteins in the time-series cohort. **C** The ratio of the abundance of human-derived proteins and microbial-derived proteins at different time points. **D** The correlation of the abundance of human-derived proteins or microbial-derived proteins between interindividuals and intraindividuals in the time-series cohort. **E** Correlation coefficient of the abundance of human-derived proteins (left) and microbial-derived proteins (right) in the time-series cohort

To investigate the stability of the tongue-coating proteome over time, we designed a time-series cohort with temporal longitudinal sampling. These samples were donated by 5 healthy volunteers and collected on days 0, 3, 6, and 9, with three samples obtained on each occasion. One volunteer did not participate in the last sampling due to oral bleeding. A total of 57 samples were finally included in the time-series cohort (Fig. 2A).

A total of 12,839 (90.19%) microbial-derived proteins and 1396 (9.81%) human-derived proteins were identified (Fig. 2B). We observed that the ratio of protein intensity derived from human versus microbiota in tongue coating fluctuates over time (Fig. 2C). This variation can be ascribed to the instability of both human and microbiota protein intensities. Within an individual, human proteins exhibited a stronger correlation than microbial proteins (r_Spearman_ human 0.968 ± 0.012, microbial 0.896 ± 0.040) over time (Fig. 2D–E). Additionally, at the phylum level, we observed a slightly lower degree of stability within an individual over time (r_Spearman_ phylum 0.839 ± 0.085) (Supplemental Fig. 3A–B). This variation may be attributed to the uneven distribution of microorganisms on the tongue and the randomness of the sampling location. Nevertheless, we observed that microbial functional COGs within an individual exhibited remarkable stability over time (r_Spearman_ intra-individual 0.959 ± 0.017) (Supplemental Fig. 3C–D).

Among different individuals, human proteins still exhibited a high level of consistency (r_Spearman_ human 0.968 ± 0.012, Fig. 2D–E). Taxonomic results are consistent with those of intra-individual (r_Spearman_ phylum 0.838 ± 0.089, Supplemental Fig. 3A–B). However, microbial proteins and COG displayed variability among different individuals (r_Spearman_ protein 0.896 ± 0.040, COG 0.831 ± 0.089, Fig. 2 D–E, Supplemental Fig. 3C–D). Therefore, microbial proteins may serve as a reliable indicator for characterizing an individual, given their temporal stability and variability among individuals.

### Functional characteristics of the tongue-coating proteome

**Fig. 3:**
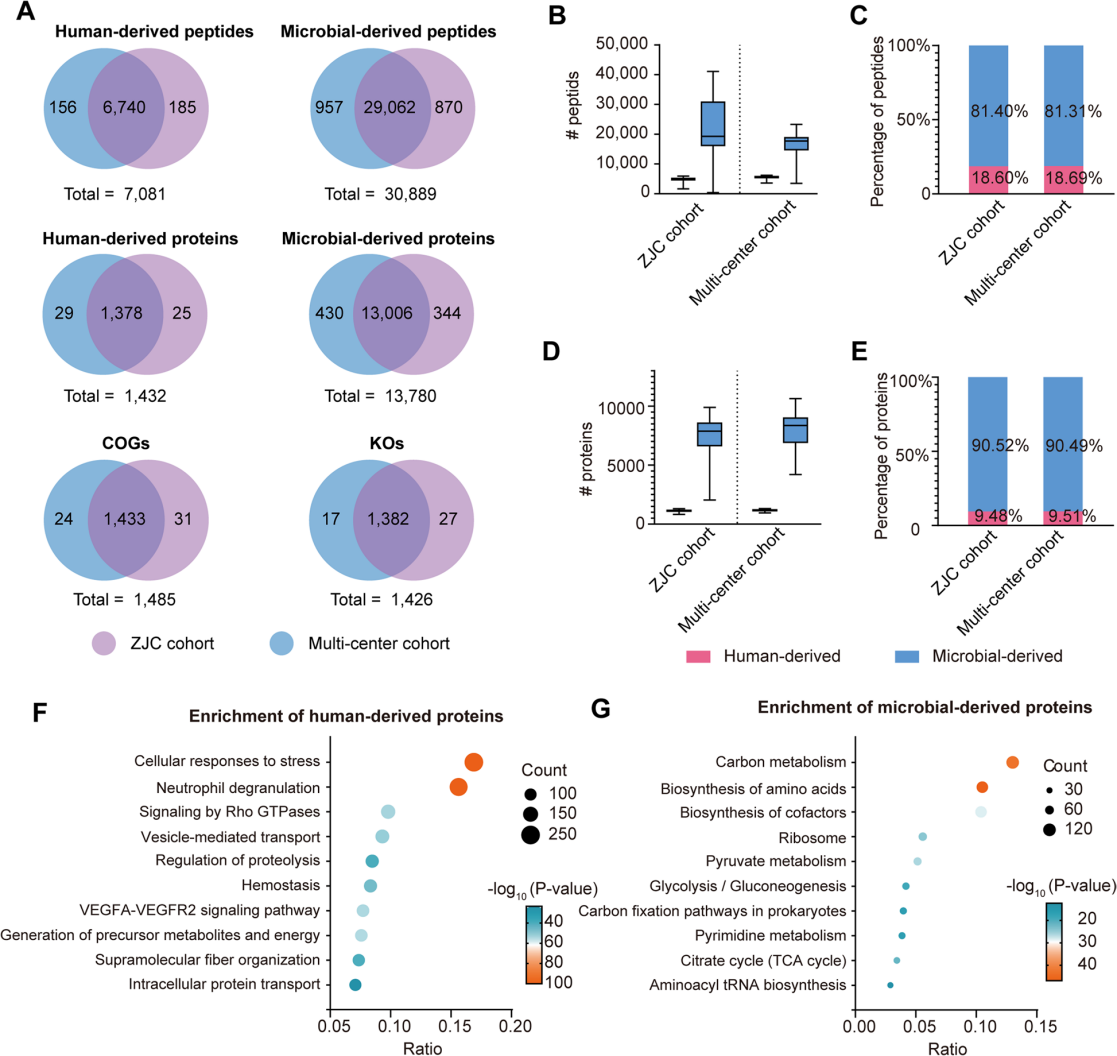
Functional characteristics of the tongue-coating proteome. **A** Comparison of identified human-derived peptides, human-derived proteins, microbial-derived peptides, microbial-derived proteins, COGs, or KOs in the ZJC cohort and multicentre cohort. **B** The number of human-derived peptides and microbial-derived peptides identified in the ZJC cohort and multi-center cohort. **C** The proportion of human-derived peptides to microbial-derived peptides in the ZJC cohort and multi-center cohort. **D** The number of human-derived proteins and microbial-derived proteins identified in the ZJC cohort and multi-center cohort. **E** The proportion of human-derived proteins to microbial-derived proteins in the ZJC cohort and the multi-center cohort. **F**–**G** Functional enrichment analysis of human-derived proteins (**F**) and microbial-derived proteins (**G**) of the ZJC cohort

Next, we compared the proteomic data of the ZJC cohort and the multi-center cohort. At the peptide level, a total of 37,970 peptides were identified in the two cohorts. A total of 7082 peptides were derived from humans, of which 6740 (95.2%) were identified in both cohorts. There were 30,889 peptides from microbiota, of which 29,062 (94.1%) were identified in both cohorts (Fig. 3A). For each sample, 4866 ± 598 human peptides and 15,298 ± 3,768 microbial peptides were identified in the ZJC cohort, while 5496 ± 535 human peptides and 16,652 ± 3536 microbial peptides were identified in the multi-center cohort (Supplemental Fig. 4A, C). In both cohorts, the number of identified microbial peptides was approximately four times higher than that of the human peptides (Fig. 3B–C).

At the protein level, a total of 15,212 proteins were identified in the two cohorts. Of these, 1432 proteins were of human origin, and 1378 (96.2%) were shared between the two cohorts. The remaining 13,780 proteins were of microbial origin, and 13,006 (94.4%) were shared between the two cohorts (Fig. 3A). The number of identified microbial proteins was approximately nine times higher than that of the human proteins (Fig. 3D–E). For each sample, 1113 ± 120 human proteins and 7393 ± 1709 microbial proteins were identified in the ZJC cohort, while in the multi-center cohort, 1154 ± 115 human proteins, and 7661 ± 1827 microbial proteins were identified per sample (Supplemental Fig. 4B–C). A total of 1485 COGs and 1426 Kos were annotated, 96.5% and 96.9% of which were commonly annotated by the two cohorts. (Fig. 3A). When comparing the two cohorts, it became evident that despite individuals hailing from different cities, the peptides, proteins, COGs, and KOs present in tongue coating exhibited a high degree of similarity.

Finally, to characterize the functional pathway profiles, we performed KEGG pathway enrichment on human proteins and microbial proteins in the ZJC cohort. Human proteins were mainly enriched in pathways such as cellular response to stress and neutrophil degranulation (Fig. 3F). Microbial proteins were mainly enriched in pathways such as carbon metabolism, biosynthesis of amino acids, and biosynthesis of cofactors (Fig. 3G). We also compared the functions of the 100 highest expressed proteins and the lowest 100 proteins in the two cohorts. The highly expressed proteins in both cohorts were enriched in pathways such as ribosome, carbon metabolism, clycolysis/cluconeogenesis, and carbon fixation in photosynthetic organisms (Supplemental Fig. 6A–B). The functions of highly expressed microproteins in the two cohorts were similar. However, there is a large functional difference in the low-abundance proteins between the two cohorts (Supplemental Fig. 6C–D). This may be related to the fact that low-abundance proteins are not easily detected, resulting in a large difference in the low-abundance proteins between the two cohorts.

### Taxonomy of the tongue-coating microbiota

**Fig. 4:**
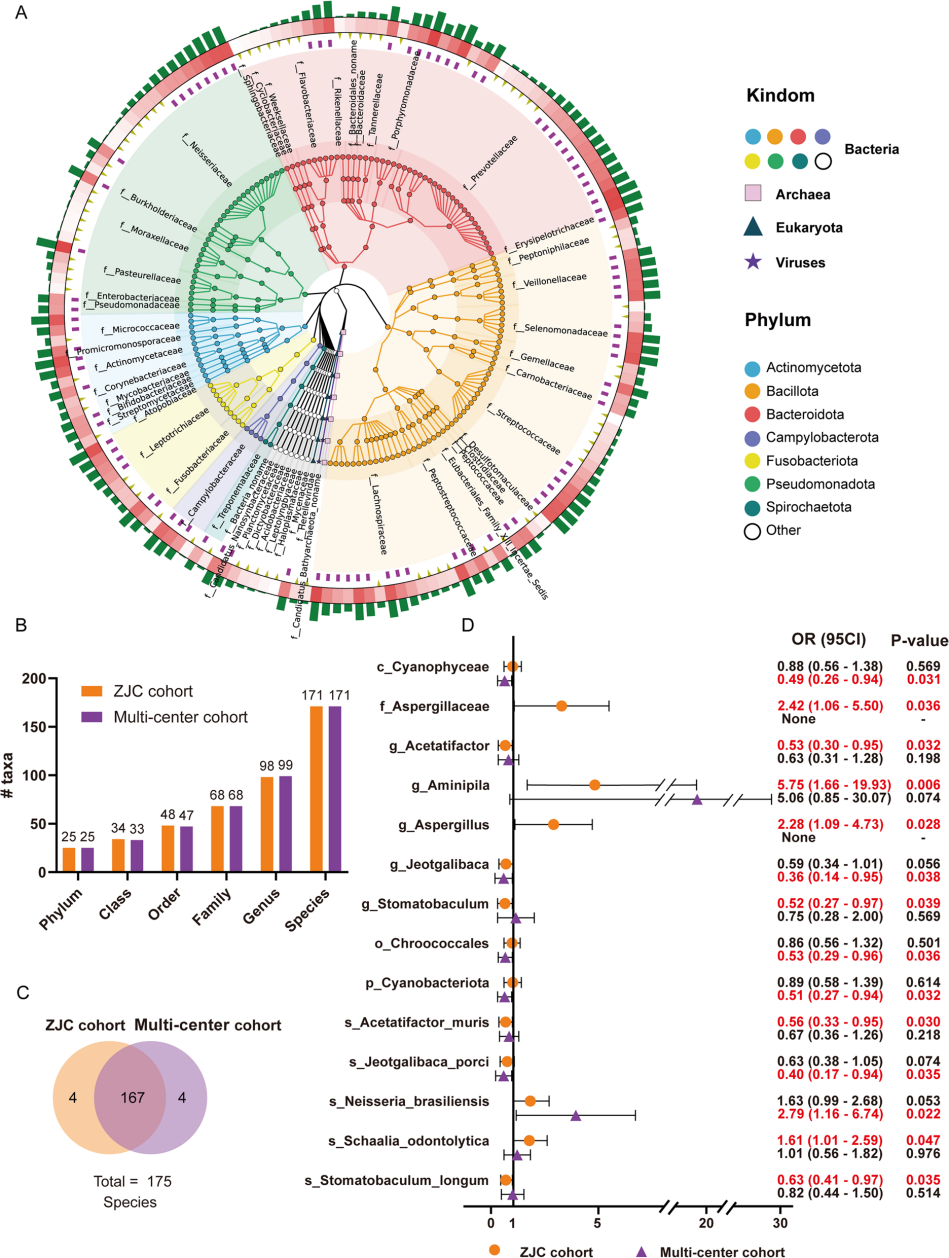
Taxonomy overview and microbiota structural alterations of tongue coating in patients with gastric cancer. **A** Taxonomic tree of the tongue-coating microbiome in the samples from the ZJC cohort. **B** The number of identified bacteria in the ZJC cohort and multi-center cohort. **C** Comparison of the total bacterial species identified in the ZJC cohort and multi-center cohort. **D** Bacteria associated with gastric cancer risk in the ZJC cohort and multi-center cohort

Using Unipept, we annotated the microbes present in all tongue-coating samples to gain a preliminary understanding of the bacterial diversity and quantity on the tongue. The tongue-coating microorganisms are mainly bacteria, with only one archaea, one eukaryote, and one virus species. Twenty-five phyla were identified in the ZJC cohort, among which Bacillota, Bacteroidota, Pseudomonadota, Actinomycetota, and Fusobacteriota included the most species (Fig. 4A). Next, we compared the bacteria identified in the ZJC cohort and the multi-center cohort. The number of bacteria identified by the multi-center cohort was similar to that in the ZJC cohort (Fig. 4B). At the species level, a total of 175 species of bacteria were identified, 167 of which were commonly identified by both cohorts (Fig. 4C). In summary, the two cohorts have similar tongue-coating microbial diversity at phylum, class, order, family, genus, and species levels (Supplemental Fig. 7).

### Microbiota structural alterations of tongue coating in patients with gastric cancer

We first compared the identified peptides, proteins, and functional profiles between gastric cancer and non-cancer groups, as well as between stage I–II and stage III–IV cancer patients in both cohorts. Similar diversity (over 95%) was found between cancer and non-cancer groups (Supplemental 5A–B), as did samples from stage I–II and stage III–IV cancer patients (Supplemental 5C–D).

Next, we conducted a regression analysis to investigate the efficacy of tongue-coating species in indicating the risk of gastric cancer (Methods). At the species level, we found that *Schaalia odontolytica* (OR (95% CI) = 1.01–2.59, *p* = 0.047) was associated with higher gastric cancer risk, while *Acetatifactor* (OR (95% CI) = 0.30–0.95, *p* = 0.032) and *Stomatobaculum longum* (OR (95% CI) = 0.27–0.97, *p* = 0.039) was associated with lower gastric cancer risk in the ZJC cohort (Fig. 4D). In the multi-center cohort, *Neisseria brasiliensis* (OR (95% CI) = 1.16–6.74, *p* = 0.022) was found to be a risk factor for gastric cancer. And *Jeotgalibace porci* (OR (95% CI) = 0.17–0.94, *p* = 0.035) was associated with lower gastric cancer risk (Fig. 4D).

### Functional changes in tongue-coating microbiota in gastric cancer patients

**Fig. 5:**
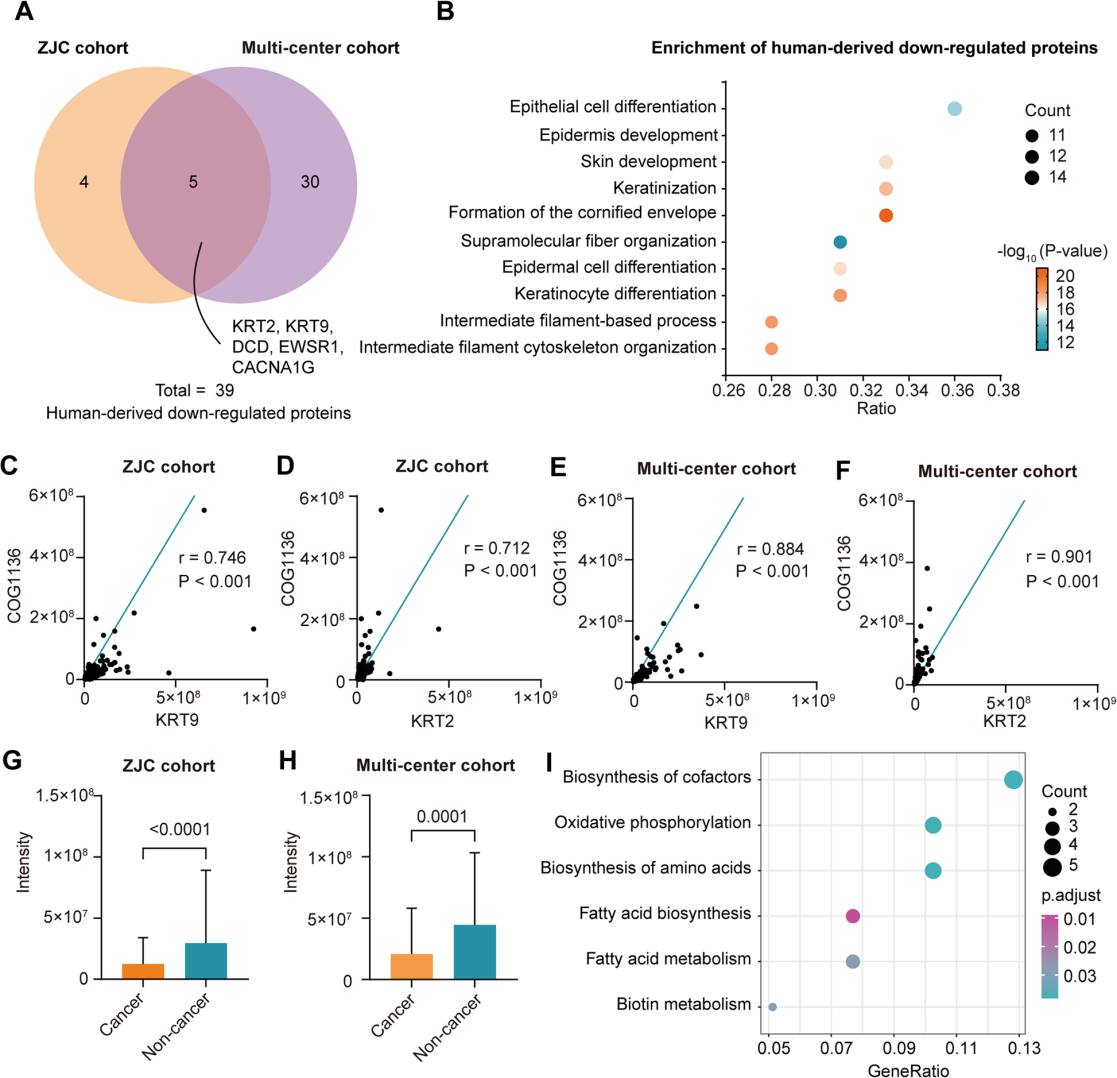
Functional changes in the microbiota of the tongue coating in gastric cancer patients. **A** Human-derived downregulated proteins in the ZJC cohort and the multi-center cohort. **B** Functional enrichment analysis of human-derived downregulated proteins in the ZJC cohort and the multi-center cohort. **C**–**F** The abundance correlation between COG1136 and KRT9 (**C**) or KRT2 (**D**) in the ZJC cohort. Abundance correlation between COG1136 and KRT9 (**E**) or KRT2 (**F**) in the multi-center cohort. **G**–**H** The intensity of COG1136 in gastric cancer and nongastric cancer samples in the ZJC cohort (**G**) and the multi-center cohort (**H**), respectively. **I** Functional enrichment analysis of microbial-derived downregulated proteins in the ZJC cohort and the multi-center cohort

To further investigate the changes in tongue-coating proteins in gastric cancer patients, we first performed differential protein analysis of human proteins in the ZJC cohort and the multi-center cohort. A total of 12 proteins were upregulated, and 9 proteins were downregulated in gastric cancer patients from the ZJC cohort (|log2 (fold change)|> 1 and *p* value < 0.05) (Supplemental Fig. 8A). In the multi-center cohort, we identified 9 upregulated proteins and 35 downregulated proteins (Supplemental Fig. 8B). A total of 5 proteins, including KRT2, KRT9, DCD, EWSR1, and CACNA1G, were downregulated in gastric cancer patients in both cohorts (Fig. 5A). KRT2 and KRT9 are crucial keratins that comprise the tongue coating [34]. Next, we performed functional enrichment of all downregulated human proteins of the tongue coating in gastric cancer patients. We found that these proteins were mainly related to the growth and differentiation of tongue epithelium, which meant that the physical barrier on the tongue surface of gastric cancer patients was weakened (Fig. 5B).

To investigate the interaction between human proteins and microbial proteins, we performed a Spearman correlation analysis between the abundance of KRT2, KRT9, and all identified COGs in the two cohorts. Both KRT2 and KTR9 were highly positively correlated with COG1136 (Fig. 5D–F). COG1136 was expressed at significantly lower levels in the tongue coating of gastric cancer patients than in that of healthy volunteers in both cohorts (Fig. 5G–H, Supplemental Fig. 8C–D). COG1136 is a class of proteins that constitute the ABC transporter, which is involved in cell protection and excreting endogenous compound production during cell metabolism and resisting environmental toxins, acting as a protective mechanism for bacteria [35–37]. The decrease in these proteins in the tongue coating of patients with gastric cancer suggested the decreased defense ability of the lingual mucosa.

Finally, we conducted KEGG pathway enrichment for the down-regulated and upregulated microbial proteins in gastric cancer patients. In gastric cancer patients, the downregulated microbial proteins were mostly enriched in the biosynthesis of cofactors, oxidative phosphorylation, and biosynthesis of amino acid pathways (Fig. 5I). The two-component system, carbon metabolism, and butanoate metabolism pathways were upregulated in gastric cancer patients (Supplemental Fig. 8E).

### A gastric cancer screening model based on microbial-derived tongue-coating proteins

**Fig. 6:**
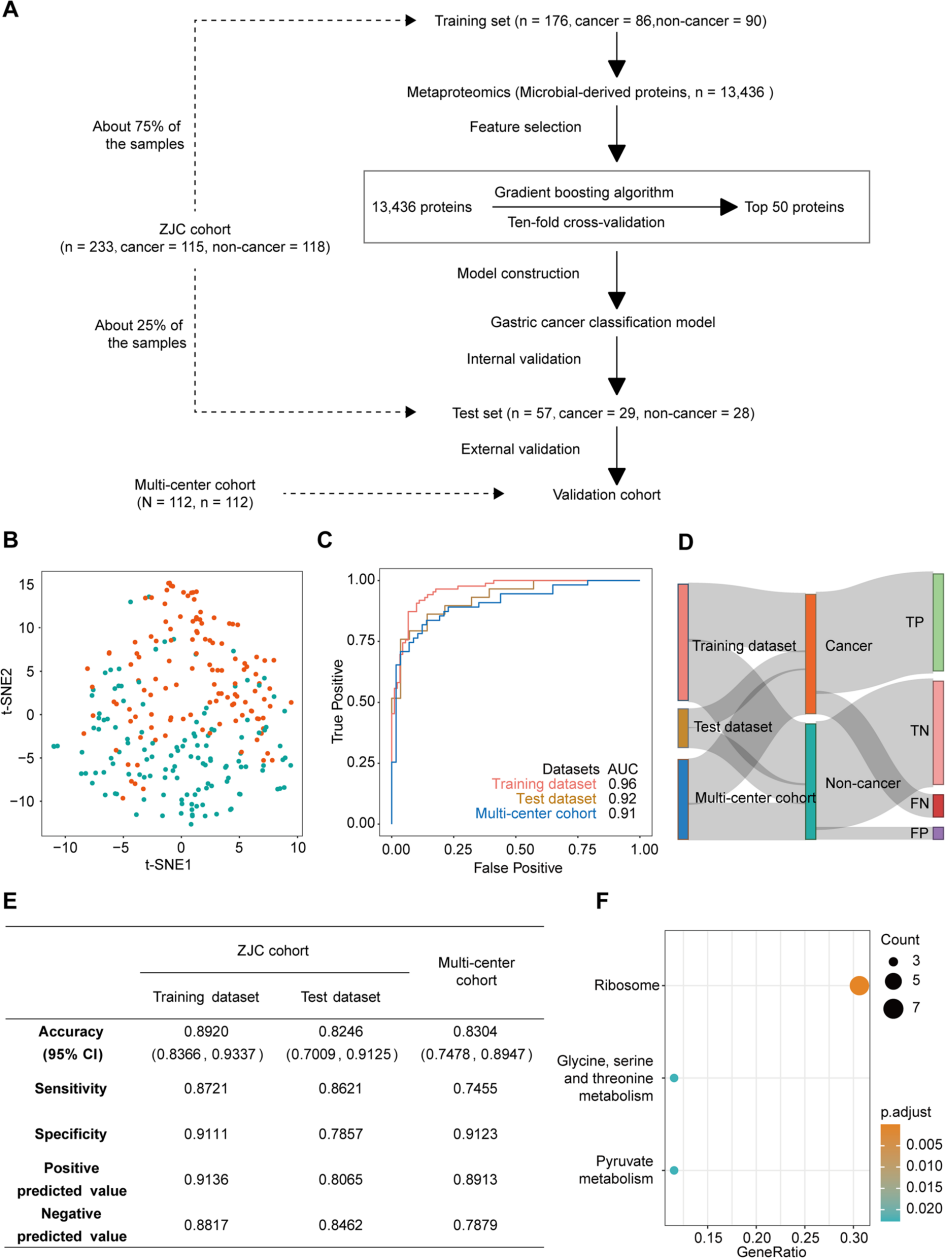
A gastric cancer screening model based on microbial-derived tongue-coating proteins. **A** Construction workflow of the gastric cancer screening model based on microbial-derived tongue-coating protein. **B** The t-distributed stochastic neighbor embedding (t-SNE) visualization of gastric cancer and nongastric cancer samples in the training dataset based on the top 50 tongue-coating microbial proteins selected by the GBM model. **C** The performance of the receiver operating characteristic curve (ROC) to classify patients with gastric cancer and healthy individuals using the 50 microbial protein features in the training data, test data, and multi-center cohort. **D** The distribution ratio and correspondence between different cohorts and samples. **E** Accuracy, sensitivity, and specificity of our screening model in different datasets. **F** Functional enrichment analysis of 50 microbial-derived proteins included in the screening model

In the above analysis, we found that taxonomy-based gastric cancer indicators were not well validated in the multi-center cohort (Fig. 4D) [38]. However, the intra-individual stability and the inter-individual variability of tongue-coating microbial proteins showed the potential to identify gastric cancer patients (Fig. 2D–E). Therefore, we used microbial proteins to generate a classification model for gastric cancer. To do so, we divided the ZJC cohort into a training dataset and a test dataset at a 3:1 ratio. Then, we used the stochastic gradient boosting model (GBM) to select the most important microbial proteins in the training dataset (Fig. 6A, Methods).

The top 50 microbial proteins were selected to identify patients with gastric cancer (Supplemental Fig. 9A). The t-distributed stochastic neighbor embedding (t-SNE) analysis based on the 50 microbial proteins showed that most patients with gastric cancer have a distinct t-SNE distribution from non-cancer individuals (Fig. 6B). In addition, the Euclidean distances between any two individuals in the two cohorts were calculated. We observed that the distance between healthy controls and gastric cancer patients in stages I–II or stages III–IV was greater than the distance between healthy controls and gastritis patients (Supplemental Fig. 9B).

The model achieved 89.6% (95% CI 84.7–93.5%) accuracy, 87.1% sensitivity, 91.92% specificity, and a 0.96 AUC in the classification of gastric cancer patients and healthy individuals in the training dataset. Furthermore, these 50 microbial proteins achieved 81.3% (95% CI 69.5–89.9%) accuracy, 83.8% sensitivity, 78.8% specificity, and 92% AUC in the internal test dataset. Finally, we tested the performance of the features in the independent validation cohort (multi-center cohort). These microbial proteins achieved 81.1% (95% CI 73.3–87.4%) accuracy, 72.6% sensitivity, 91.5% specificity, and an 87% AUC in the independent cohort (Fig. 6C–E, Supplemental Fig. 9C–E). These results demonstrated that the microbial proteins of the tongue coating can be used as effective biomarkers in gastric cancer patient classification.

KEGG enrichment suggested that these 50 proteins were mostly associated with ribosomes, a multiunit complex that converts mRNA into proteins and plays a critical role in cell proliferation, differentiation, apoptosis, development, and transformation [39]. The changes in microbial ribosome proteins in the tongue coating of patients with gastric cancer suggested important changes in microbial protein synthesis.

## Discussion

In our previous study, we found that the tongue coating of gastric cancer patients was notably thicker than that of healthy individuals [10]. The underlying causes of this distinction remain inadequately elucidated. There is some indication that microbes may hold a pivotal role in accounting for this variation [19, 40, 41]. The oral cavity is the second largest microbial ecosystem in the human body, with more than 700 species and phylotypes [42]. Both traditional wisdom and contemporary scientific investigations have confirmed that shifts in the equilibrium of oral bacterial composition might serve as indicators of pathological transformations. These transformations encompass not only oral disorders like halitosis, dental caries, and periodontitis but also systemic conditions affecting the respiratory system [43], circulatory [44], endocrine systems [45], and even malignancies [46].

Most previous studies characterized tongue coating at the genomic level based on metagenomics [19, 20]. Nevertheless, proteins play a crucial role in biological function. With the advent of metaproteomics, researchers are now able to analyze the contribution of microorganisms in disease processes at the protein level. Metaproteomics provides the opportunity to quantify protein intensity, elucidate biodiversity, and understand biological functions at the protein level. Additionally, the joint analysis of microbial and human proteomes can shed light on the interplay between microbes and hosts.

Quantitative proteomic characterization of the tongue-coating microbiome is particularly difficult due to the ultrahigh complexity of the samples. In our experiment, we employed the PCT-DIA method to identify and quantify both human proteins and microbial proteins in tongue-coating samples. This allowed us to elucidate the protein-level factors contributing to changes in tongue coating observed in gastric cancer patients. Given the possibility of specific microorganisms in the tongue coating of gastric cancer patients, we performed metagenomic sequencing on a subset of samples within the training set to establish a comprehensive database. To ensure the broadest sample coverage, we included healthy individuals, chronic gastritis patients, and those with both stage I–II and stage III–IV gastric cancer in our sample collection. This approach allowed us to create a comprehensive database encompassing various sample types. Furthermore, we combined the human proteins from the UniProt database with our tongue-coating microbial protein database to ensure precise peptide classification during the library search. This comprehensive approach led to the identification of a total of 1432 human-derived and 13,780 microbial-derived proteins. In another proteomic study conducted by Rabe et al., a total of 1857 human proteins and 3969 bacterial proteins were identified in 24 tongue coating and saliva samples from healthy individuals using Data-Dependent Acquisition (DDA) technology [11]. Based on the number of identified proteins, particularly the microbial-derived proteins, our method appears to offer distinct advantages. Hence, employing metagenomic sequencing to build a protein database and combining it with the PCT-DIA method for the study of tongue-coating proteomics is a viable approach.

Previous studies have not addressed the stability of tongue-coating proteins. Therefore, we designed a time-series cohort with continuous sampling and discovered that tongue-coating proteins remain stable over time within the same individual. This stability extends to the functionality of microbial proteins. Moreover, the lower correlation in microbial protein intensity among different individuals suggests that microbial-derived proteins exhibit significant variability between individuals, making them promising markers for individual identification.

To delve deeper into the characteristics of tongue-coating proteins in gastric cancer patients, we conducted a comparative analysis of the proteomic data from the ZJC cohort and the multi-center cohort. Surprisingly, despite the diverse geographical origins of these cohorts, we observed a high degree of diversity similarity in terms of peptides, proteins, COGs, and KOs. Additionally, when we compared tongue-coating protein characteristics between gastric cancer and non-cancer samples, as well as among samples with different cancer stages, we again found substantial similarities. Therefore, when examining protein profiles alone, the proteins present in tongue coating across various groups appear highly alike, presenting a challenge for differentiation.

Protein functional analysis revealed that human-derived proteins are predominantly associated with pathways like “cellular response to stress” and “neutrophil degranulation.” The tongue’s microenvironment is notably intricate, subject to a variety of stimuli such as mechanical forces, temperature fluctuations, and the presence of microorganisms [47]. The activation of these pathways may be linked to the external pressure on the lingual mucosa. On the other hand, microbial-derived proteins are predominantly associated with pathways like “carbon metabolism,” “biosynthesis of amino acids,” and “biosynthesis of cofactors,” which play crucial roles in microbial growth and metabolism.

In the bacterial profile analysis, *Schaalia odontolytica* and *Neisseria brasiliensis* were detected as the indicators of gastric cancer risk in the ZJC cohort and the multi-center cohort, respectively. *Schaalia odontolytica* and *Neisseria brasiliensis* belongs to the genus *Schaalia* and *Neisseria*, respectively. *Schaalia* and *Neisseria* are the major NO2^−^-producing bacteria in our oral [48], which can convert ammonia into nitrite and N-nitroso compounds. The elevated levels of nitrite or N-nitroso compounds may contribute to the development of gastric cancer [49]. In addition, Herreros-Pomares et al. reported that *Schaalia odontolytica* is enriched in the oral cavity of patients with proliferative verrucous leukoplakia (PVL), a high-risk precancerous lesion that easily develops into oral squamous cell carcinoma [50]. Xu et al. reported that *Schaalia* is a high-risk factor for young-onset colorectal cancer patients [51]. Li et al. found that *Neisseria* was significantly increased in the saliva of esophageal adenocarcinoma patients [52]. However, in this study, *Schaalia odontolytica* and *Neisseria brasiliensis* could not be mutually verified in the two cohorts. It is not unusual for different studies to report inconsistent or even contradictory bacterial spectra [20], and regional disparities play a significant role in contributing to this outcome [38]. The ZJC cohort was predominantly collected from coastal cities in eastern China, while a substantial portion of the samples in the multi-center cohort originated from the central region of China. These areas vary in terms of dietary and lifestyle habits. Consequently, regional disparities could introduce biases if disease analysis is solely reliant on the bacterial spectrum. Remarkably, the genus *Aminipila* demonstrated the most notable odds ratio (OR) for gastric cancer, registering at 5.75 in the ZJC cohort and 5.06 in the multi-center cohort. *Aminipila* has not been previously investigated in the context of cancer studies, but our data indicate that it warrants further exploration.

In our analysis of human-derived proteins in tongue coating, we observed a significant downregulation of KRT2, KRT9, and DCD in the tongue coating of gastric cancer patients. KRT2 and KRT9 are essential keratins that constitute the structural components of the tongue coating [34]. The reduction in keratin content within the tongue coating diminishes the physical barrier effect of the tongue surface [53–55]. Reduced keratin levels can lead to dyskeratosis of the tongue coating, causing it to accumulate and become thicker in patients with gastric cancer compared to healthy individuals. DCD is a naturally occurring polypeptide with potent antibacterial activity against *E. coli*, *E. faecalis*, *S. aureus*, and *C. albicans* [56]. The reduction in DCD levels within the tongue coating implies reduced bacterial resistance in patients with gastric cancer. Downregulation of KRT2, KRT9, and DCD collectively indicates a decreased microbial resistance in the oral cavity of gastric cancer patients. Correspondingly, we observed a significant positive correlation between the abundance of microbial ABC transporters and keratin. Notably, microbial-derived proteins linked to ABC transporters exhibited a substantial reduction in the tongue coating of gastric cancer patients. ABC transporters are recognized as crucial elements that enable bacteria to withstand adverse environments [35–37]. We speculate that the decreased bacteriostatic ability of the oral cavity of patients with gastric cancer reduces the survival pressure of bacteria, which leads to the downregulation of ABC transporter expression. Previously, Bobes et al. reported that human-derived ABCC4 and ABCG2 are highly expressed in proliferating keratinocytes [57], but no research has reported the effect of microbial-derived ABC transporters on human epidermal keratinization. Our findings may offer new insights into the interaction between microorganisms and the human oral epithelium.

Gastric cancer is characterized by a poor prognosis, limited treatment options, and a tendency for metastasis, recurrence, and drug resistance. There is a critical need for a dependable tool for early gastric cancer screening. While endoscopy is considered the gold standard for gastric cancer screening, it is a relatively costly and invasive procedure associated with varying degrees of patient discomfort. Clinical markers such as carcinoembryonic antigen (CEA), carbohydrate antigen (CA) 199, CA724, CA125, CA242, pepsinogen, and alpha-fetoprotein are commonly used for gastric cancer screening. Nevertheless, these markers exhibit low specificity and sensitivity and lack the required specificity. Leveraging the stability of microbial-derived proteins within individuals and their significant variation between individuals in tongue coating, we developed a gastric cancer screening model based on 50 microbial proteins. This model demonstrated an accuracy of 81.1% (95%CI 73.3–87.4%), with 72.6% sensitivity, 91.5% specificity, and an impressive 87% AUC in the independent validation cohort. In comparison to gastroscopy, this tongue-coating protein-based screening method is non-invasive, offers convenient sampling, and overcomes the limitations of gastroscopy, which necessitates specialized equipment and expert medical personnel. Moreover, when compared to serum pepsinogen detection, it exhibits higher specificity.

This method has the potential for large-scale application. We have discovered that tongue images and proteins can be used to identify gastric cancer. To validate these findings on a broader scale, we have launched a large prospective clinical study involving 20,000 tongue-coating samples (NCT05794841).

## Conclusions

Our study has developed a quantitative tongue-coating proteomics method based on PCT-DIA. We identified distinctive changes in tongue-coating proteins among patients with gastric cancer and built a highly precision gastric cancer screening model using microbial-derived tongue-coating proteins. Our research represents a step towards a potential noninvasive biomarker for gastric cancer that is objective and suitable for long-term monitoring. Nonetheless, further investigation is required to uncover the influence of dietary habits and geographic disparities before deploying tongue-coating protein profiling as a biomarker for larger cohorts. Moreover, in more complex clinical contexts, where there is a substantial overlap of microbial proteins across different diseases, distinguishing disease-specific alterations from the identified tongue-coating proteins becomes a formidable task. Therefore, we look forward to future research endeavors aimed at addressing this challenge.

### Supplementary Information

**Additional file 1:** **Supplemental Figure 1. **Tongue coating spectral library construction and its characteristics. A  Construction workflow of the tongue coating protein database. B Construction workflow of the tongue coating protein spectral library. C The entry composition of the tongue coating spectral library. D The distribution of peptide precursor m/z. E The counts of the different charge states of peptide precursors. F The distribution of the lengths of identified peptides. G The numbers and their corresponding ratios of proteotypic peptides for each protein. H Ion counts of each fragment type. 

**Additional file 2:** **Supplemental Figure 2. **Quality control of the tongue coating proteome. A. Batch design for the processing of tongue coating samples in the ZJC cohort and the Multi-center cohort. B. Pearson correlation analysis of technical replication samples in the ZJC cohort. C. Pearson correlation analysis of technical replication samples in Multi-center cohort. D. Pearson correlation analysis of pooled samples in the ZJC cohort. E. Pearson correlation analysis of pooled samples in Multi-center cohort.

**Additional file 3:** **Supplemental Figure 3. **Correlation coefficient of the microbial taxonomy and protein function.A. Correlation of the microbial taxonomy at phylum level ; B. The correlation coefficient of the microbial taxonomy between interindividuals and intraindividuals in the time-series cohort; C. Correlation of the microbial-derived proteins function (COGs) in the time-series cohort; D. The correlation coefficient of the COGs between interindividuals and intraindividuals in the time-series cohort.

**Additional file 4:** **Supplemental Figure 4. **Number of peptides and proteins identified in the ZJC cohort and Multi-center cohort. A. Number of peptides and proteins identified in the ZJC cohort; B. Number of peptides and proteins identified in the Multi-center cohort; C. Comparation of the number of peptides and proteins identified in the ZJC cohort and Multi-center cohort.

**Additional file 5:** **Supplemental Figure 5. **Comparison of human-derived peptides, human-derived proteins, microbial-derived peptides, microbial-derived proteins, COGs, and KOs in cancer and noncancer patients in the ZJC cohort (A) and the Multi-center cohort (B). Comparison of human-derived peptides, human-derived proteins, microbial-derived peptides, microbial-derived proteins, COGs, and KOs in stage I-II and stage III-IV patients in the ZJC cohort (C) and the Multi-center cohort (D).

**Additional file 6:** **Supplemental Figure 6. **Functional enrichment analysis of proteins with the highest and lowest expression levels. Functional enrichment of 100 microbial derived proteins with the highest expression in the ZJC cohort (A) and the Multi-center cohort (B). Functional enrichment of 100 microbial derived proteins with the lowest expression in the ZJC cohort (C) and the Multi-center cohort (D).

**Additional file 7:** **Supplemental Figure 7. **Comparison of the identified bacteria at the phylum, class, order, family, genus, or species level in the ZJC cohort and the Multi-center cohort.

**Additional file 8: Supplemental Figure 8**. Functional analysis of differential expression of human-derived and microbial-derived tongue coating proteins. A. Volcano plot of significantly differentially expressed human-derived proteins between cancer and noncancer samples in the ZJC cohort. B. Volcano plot of significantly differentially expressed human-derived proteins between cancer and noncancer samples in the Multi-center cohort. C. Clusters of orthologous groups (COG) categories of differentially expressed microbial-derived proteins of the ZJC cohort. D. Heatmap of differentially expressed COGs in defence mechanisms. E. Functional enrichment analysis of microbial-derived upregulated proteins in the ZJC cohort and the Multi-center cohort.

**Additional file 9:** **Supplemental Figure 9.** Marker microbial-derived proteins are useful to recognize patients with gastric cancer. A. The abundance of 50 marker proteins in patients with gastric cancer and controls. B. The Euclidean distance between different individuals is shown in the heatmap. C–E. The confusion matrix of the predicted results in the training dataset (C), test dataset (D), and Multi-center cohort (E).

**Additional file 10: Supplemental File 1.**

**Additional file 11: Supplemental File 2.** Tongue coating spectral library construction and its characteristics.

**Additional file 12: Supplement Table 1.** Comparison of clinical information on cancer and non-cancer samples in the ZJC.

## Data Availability

The proteomic data reported in this paper has been deposited in the OMIX, China National Center for Bioinformation/Beijing Institute of Genomics, Chinese Academy of Sciences (https://ngdc.cncb.ac.cn/omix/release/OMIX002843). All data is available for download.

## Abbreviations

AI-based: Artificial intelligence-based
ANC: Acetonitrile
AUC: Area under the curve
CA: Carbohydrate antigen
CEA: Carcinoembryonic antigen
COG: Clusters of orthologous groups
DDA: Data-dependent acquisition
DEP: Differential protein
GBM: Gradient boosting model
GLOBOCAN: Global cancer observatory
IAA: Iodoacetamide
KO: KEGG ortholog
MS: Mass spectrometry
OR: Odds ratio
PCT-DIA: Pressure cycling technology and data-independent acquisition
PVL: Proliferative verrucous leukoplakia
SCC: Sichuan cancer hospital
TFA: Trifluoroacetic acid
t-SNE: T-Distributed stochastic neighbor embedding
WZ: Wenzhou medical university
ZJC: Zhejiang cancer hospital
ZJT: Zhejiang hospital of traditional Chinese medicine
