# Supplementary figures and images for: In-depth metaproteomics analysis of tongue coating for gastric cancer: a multicenter diagnostic research study
Source: Microbiome. 2024 Jan 8;12:6. doi: 10.1186/s40168-023-01730-8 (PMC10773145; doi:10.1186/s40168-023-01730-8)

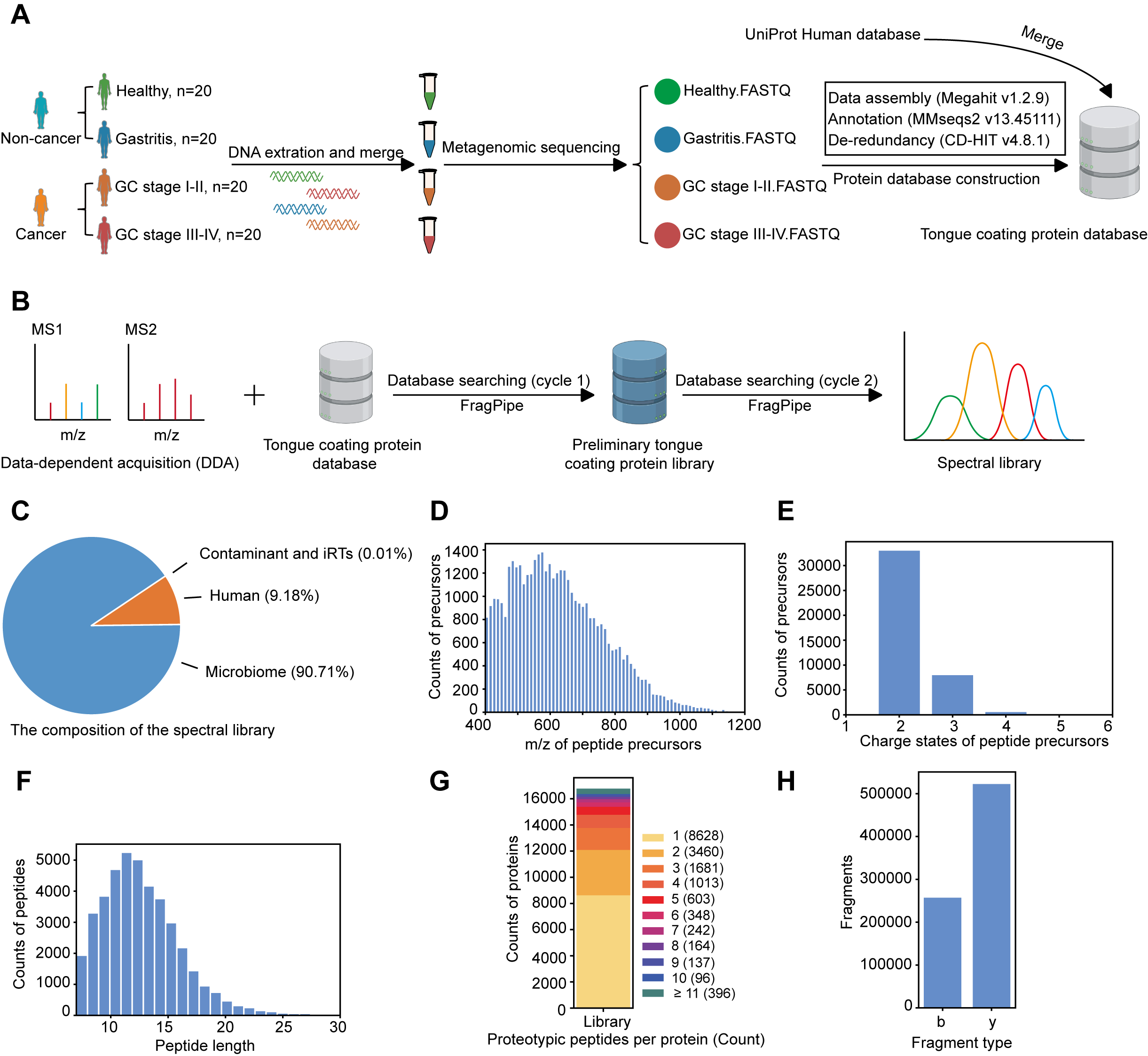

Supplement: Supplementary file 2 — Additional file 1: Supplemental Figure 1. Tongue coating spectral library construction and its characteristics. A Construction workflow of the tongue coating protein database. B Construction workflow of the tongue coating protein spectral library. C The entry composition of the tongue coating spectral library. D The distribution of peptide precursor m/z. E The counts of the different charge states of peptide precursors. F The distribution of the lengths of identified peptides. G The numbers and their corresponding ratios of proteotypic peptides for each protein. H Ion counts of each fragment type. [file 40168_2023_1730_MOESM1_ESM.tif]

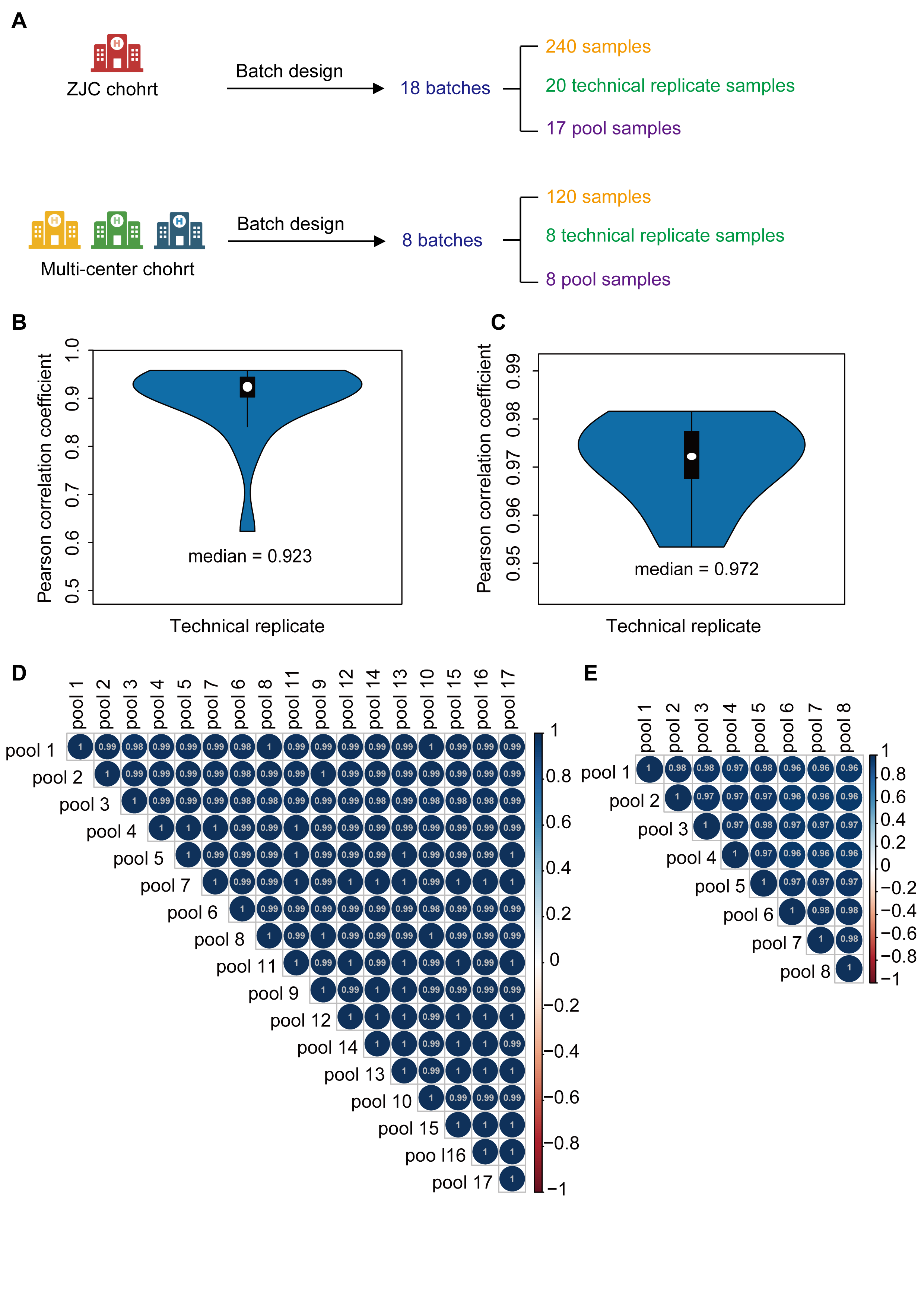

Supplement: Supplementary file 3 — Additional file 2: Supplemental Figure 2. Quality control of the tongue coating proteome. A. Batch design for the processing of tongue coating samples in the ZJC cohort and the Multi-center cohort. B. Pearson correlation analysis of technical replication samples in the ZJC cohort. C. Pearson correlation analysis of technical replication samples in Multi-center cohort. D. Pearson correlation analysis of pooled samples in the ZJC cohort. E. Pearson correlation analysis of pooled samples in Multi-center cohort. [file 40168_2023_1730_MOESM2_ESM.tif]

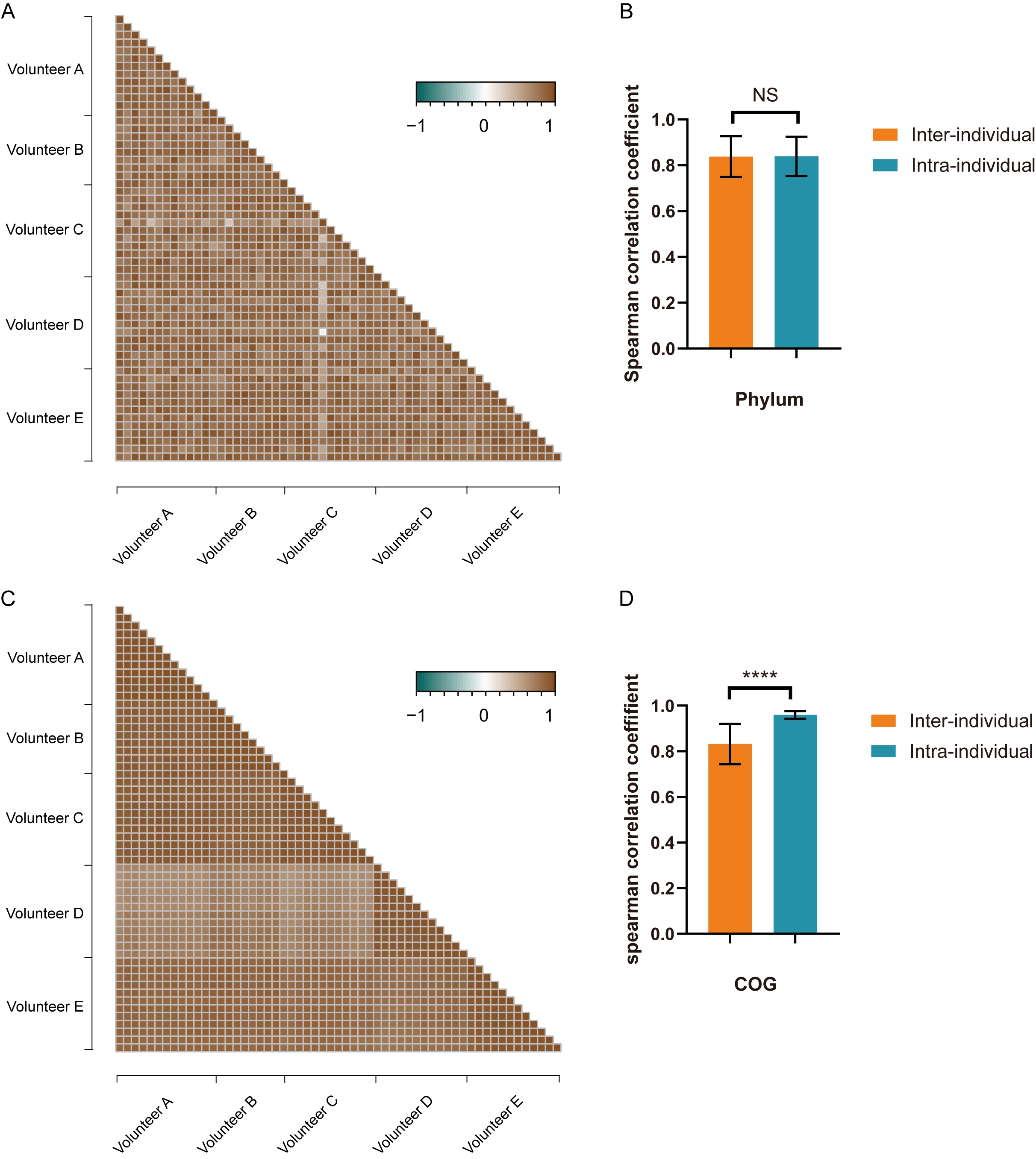

Supplement: Supplementary file 4 — Additional file 3: Supplemental Figure 3. Correlation coefficient of the microbial taxonomy and protein function.A. Correlation of the microbial taxonomy at phylum level ; B. The correlation coefficient of the microbial taxonomy between interindividuals and intraindividuals in the time-series cohort; C. Correlation of the microbial-derived proteins function (COGs) in the time-series cohort; D. The correlation coefficient of the COGs between interindividuals and intraindividuals in the time-series cohort. [file 40168_2023_1730_MOESM3_ESM.tif]

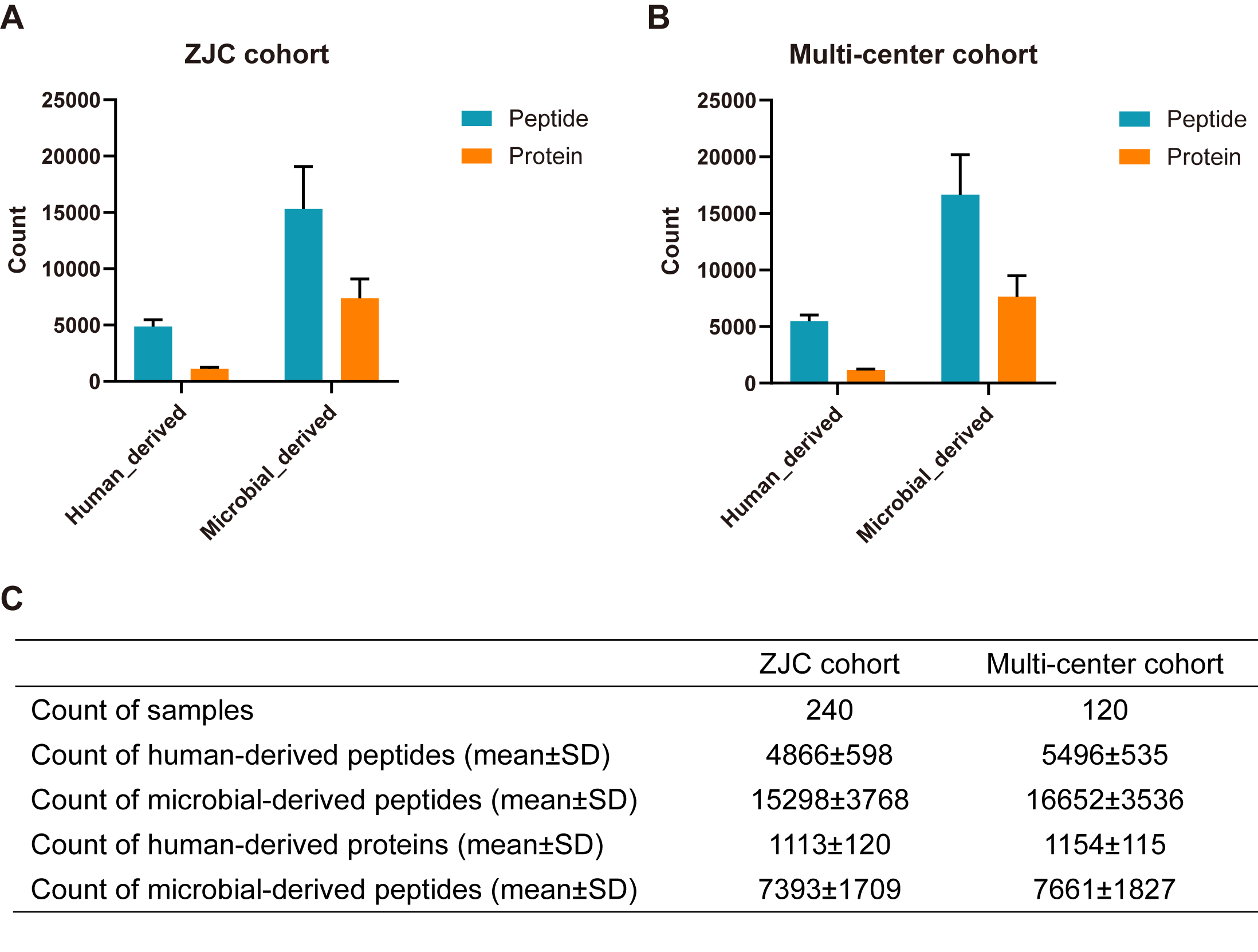

Supplement: Supplementary file 5 — Additional file 4: Supplemental Figure 4. Number of peptides and proteins identified in the ZJC cohort and Multi-center cohort. A. Number of peptides and proteins identified in the ZJC cohort; B. Number of peptides and proteins identified in the Multi-center cohort; C. Comparation of the number of peptides and proteins identified in the ZJC cohort and Multi-center cohort. [file 40168_2023_1730_MOESM4_ESM.tif]

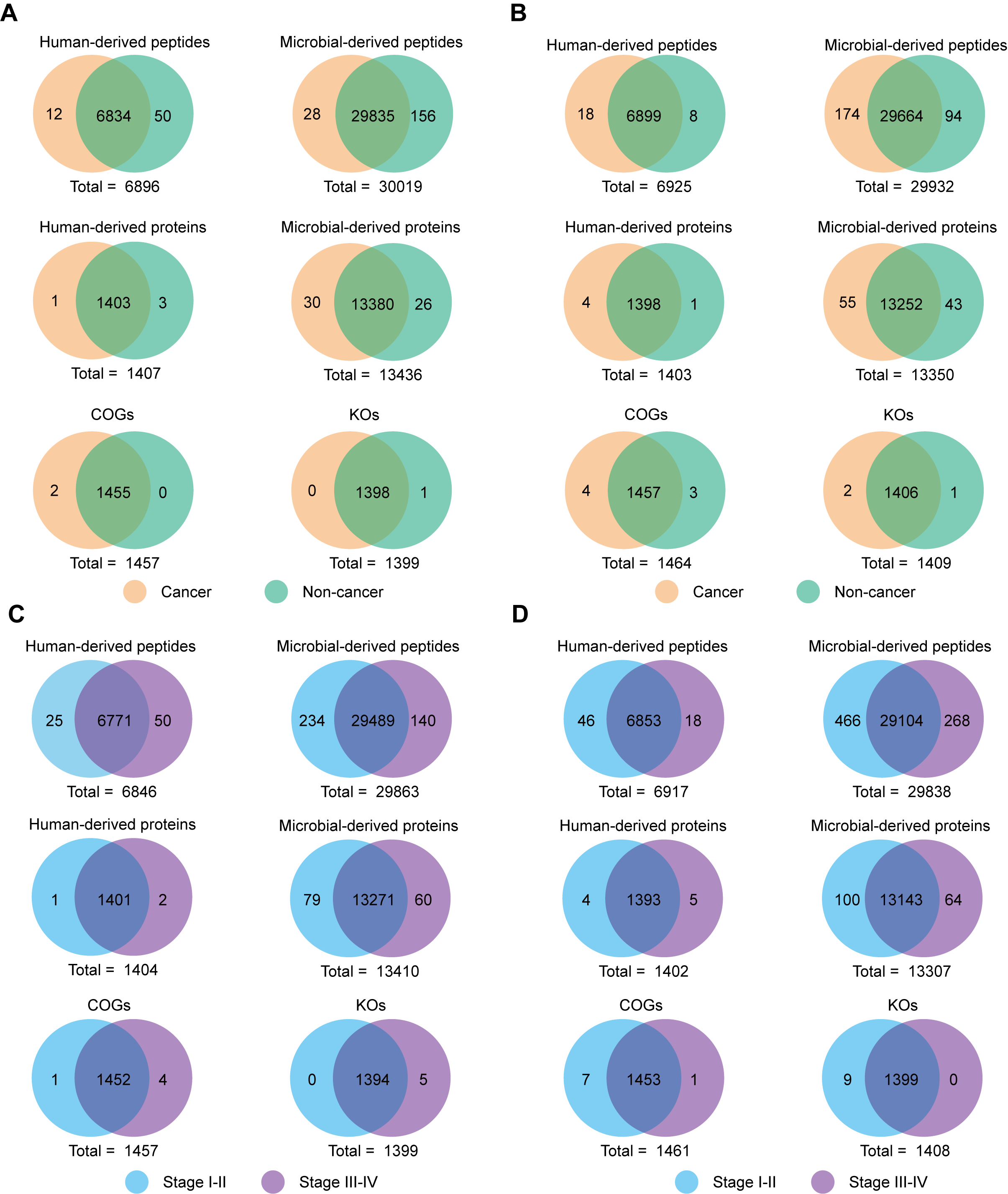

Supplement: Supplementary file 6 — Additional file 5: Supplemental Figure 5. Comparison of human-derived peptides, human-derived proteins, microbial-derived peptides, microbial-derived proteins, COGs, and KOs in cancer and noncancer patients in the ZJC cohort (A) and the Multi-center cohort (B). Comparison of human-derived peptides, human-derived proteins, microbial-derived peptides, microbial-derived proteins, COGs, and KOs in stage I-II and stage III-IV patients in the ZJC cohort (C) and the Multi-center cohort (D). [file 40168_2023_1730_MOESM5_ESM.tif]

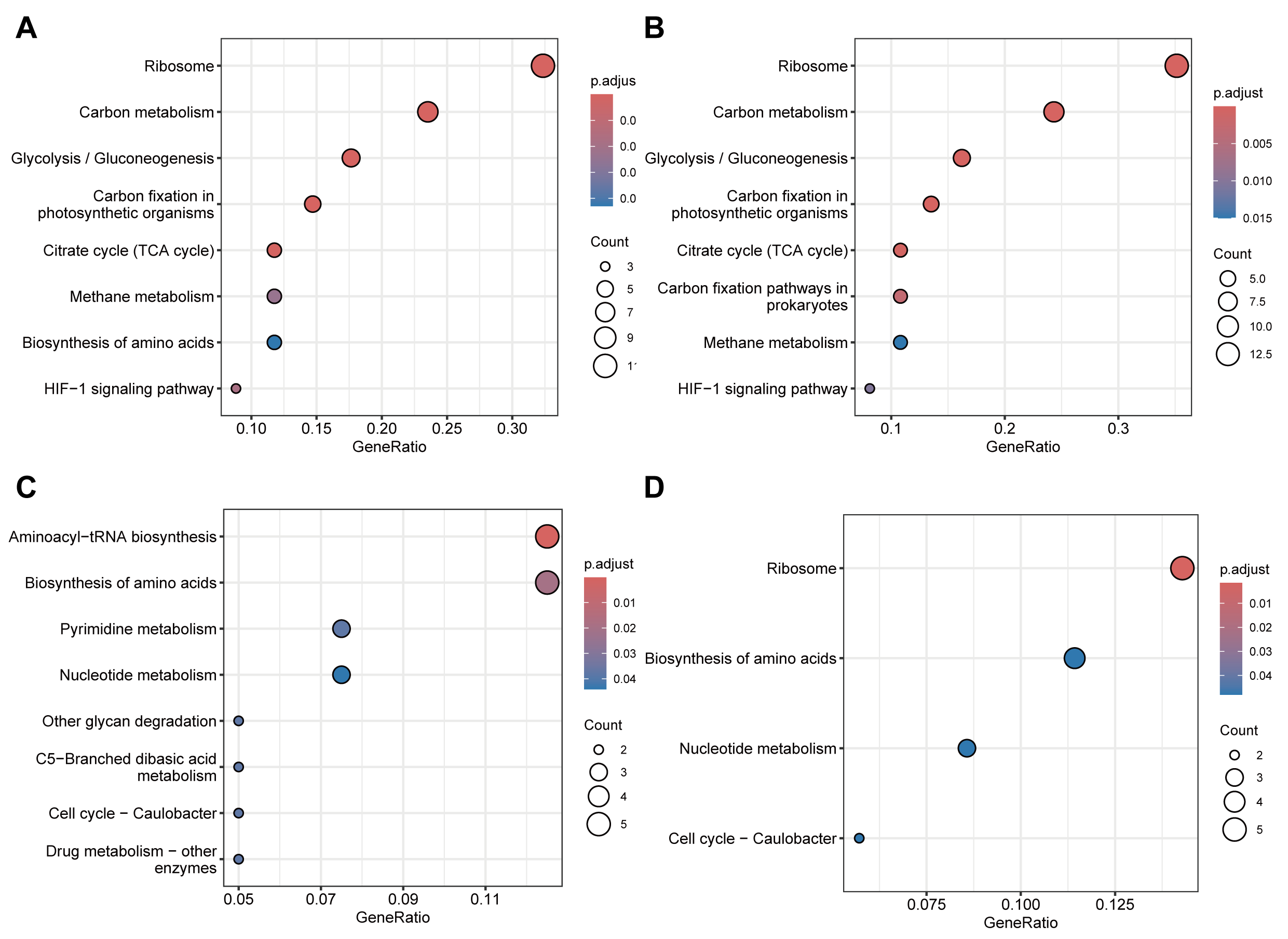

Supplement: Supplementary file 7 — Additional file 6: Supplemental Figure 6. Functional enrichment analysis of proteins with the highest and lowest expression levels. Functional enrichment of 100 microbial derived proteins with the highest expression in the ZJC cohort (A) and the Multi-center cohort (B). Functional enrichment of 100 microbial derived proteins with the lowest expression in the ZJC cohort (C) and the Multi-center cohort (D). [file 40168_2023_1730_MOESM6_ESM.tif]

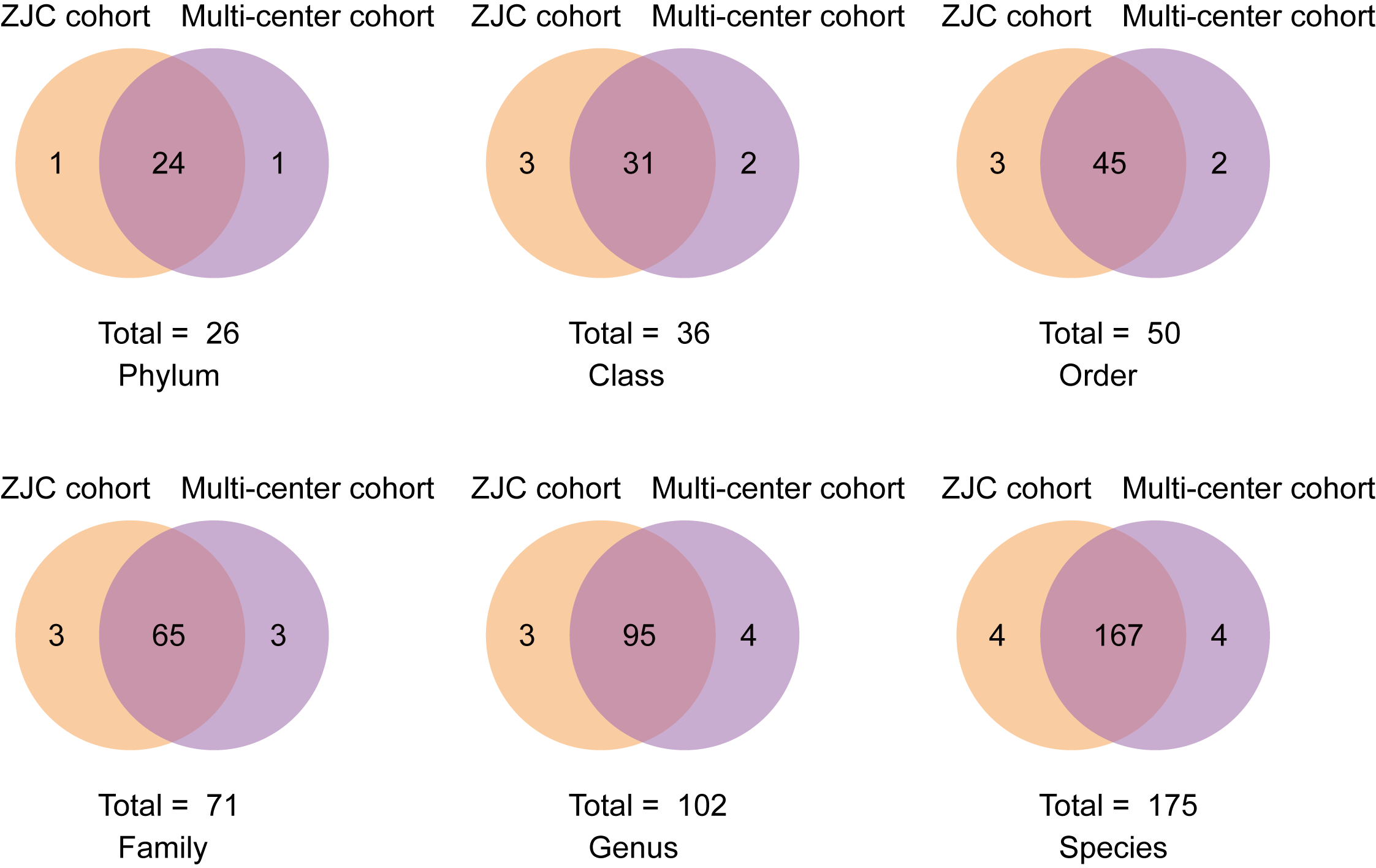

Supplement: Supplementary file 8 — Additional file 7: Supplemental Figure 7. Comparison of the identified bacteria at the phylum, class, order, family, genus, or species level in the ZJC cohort and the Multi-center cohort. [file 40168_2023_1730_MOESM7_ESM.tif]

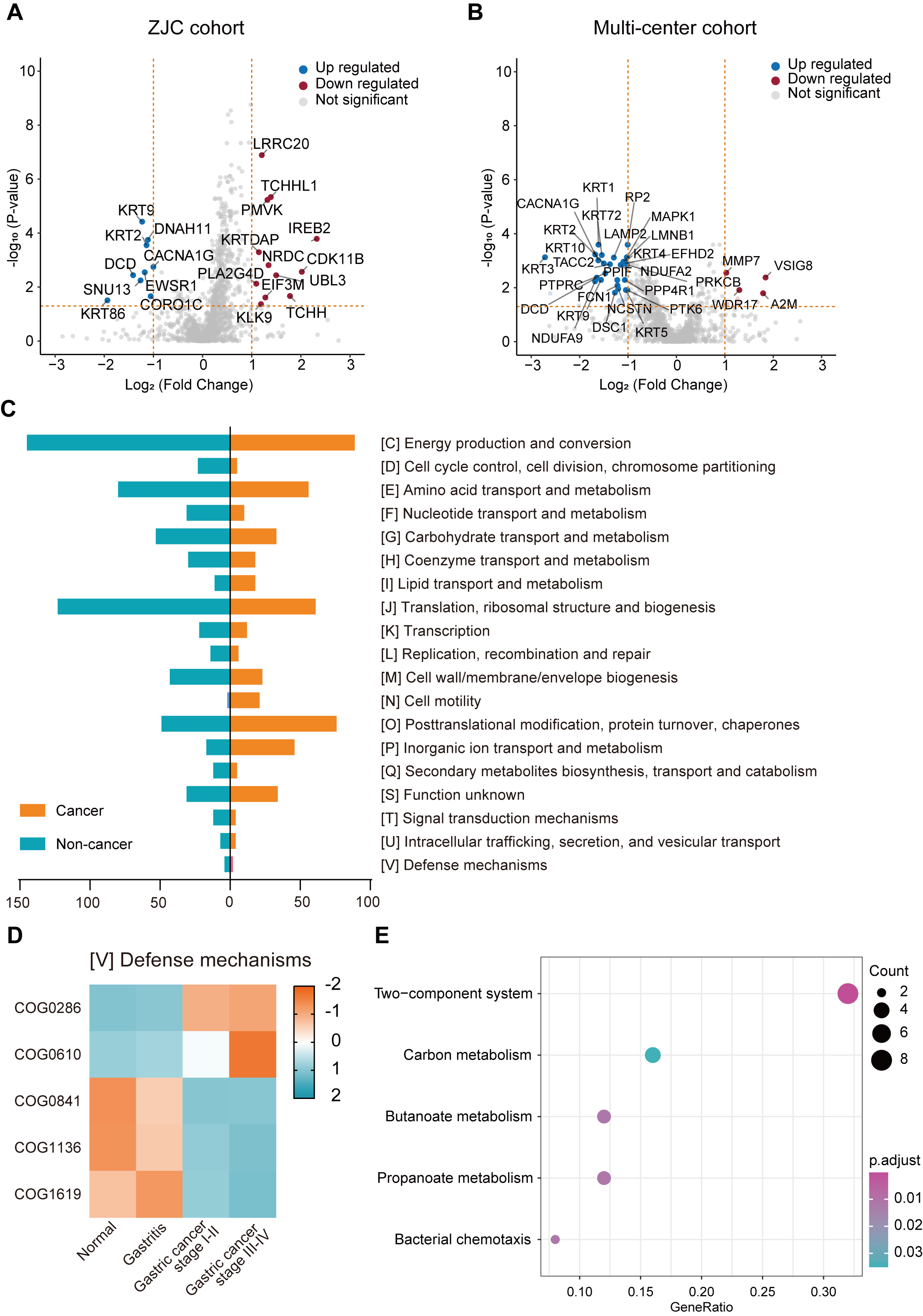

Supplement: Supplementary file 9 — Additional file 8: Supplemental Figure 8. Functional analysis of differential expression of human-derived and microbial-derived tongue coating proteins. A. Volcano plot of significantly differentially expressed human-derived proteins between cancer and noncancer samples in the ZJC cohort. B. Volcano plot of significantly differentially expressed human-derived proteins between cancer and noncancer samples in the Multi-center cohort. C. Clusters of orthologous groups (COG) categories of differentially expressed microbial-derived proteins of the ZJC cohort. D. Heatmap of differentially expressed COGs in defence mechanisms. E. Functional enrichment analysis of microbial-derived upregulated proteins in the ZJC cohort and the Multi-center cohort. [file 40168_2023_1730_MOESM8_ESM.tif]

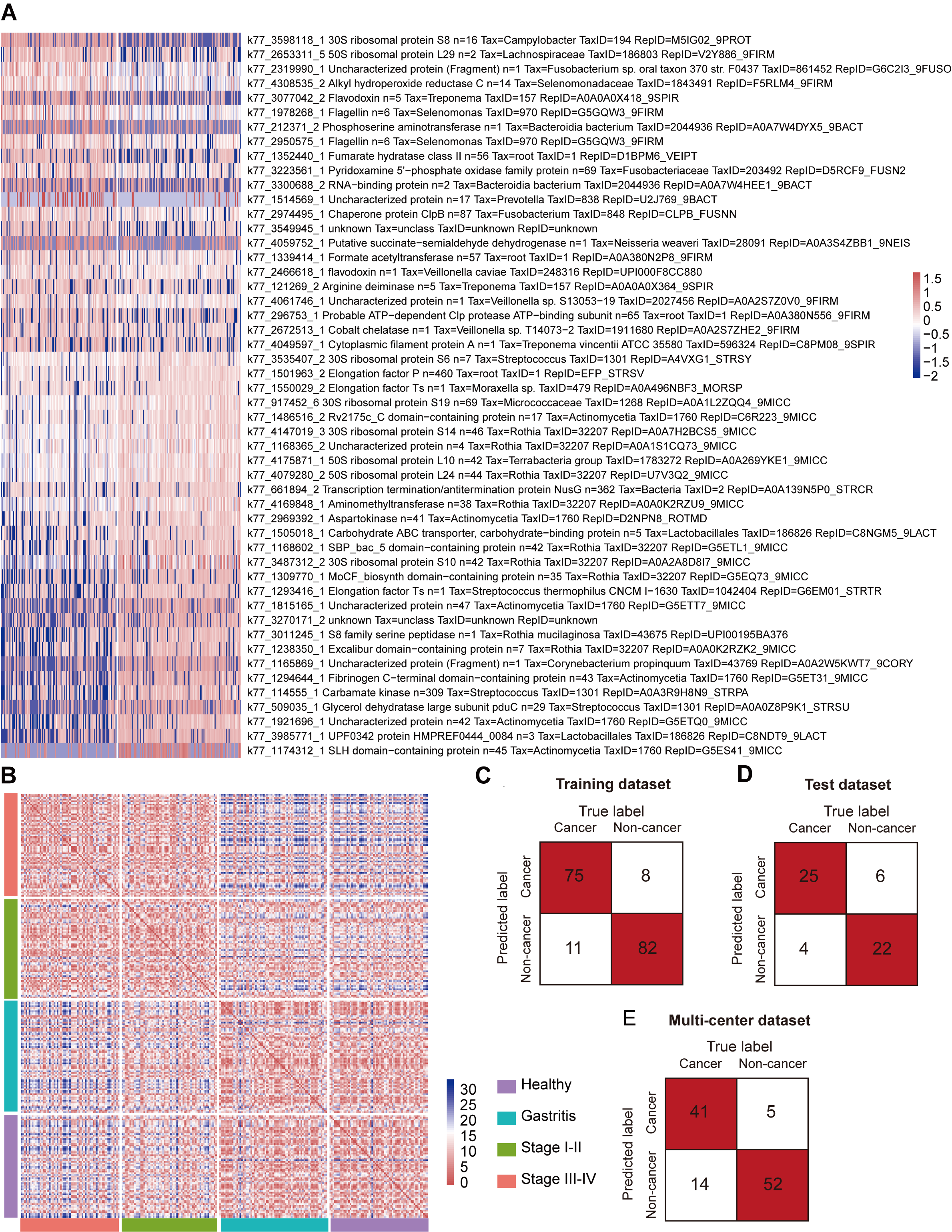

Supplement: Supplementary file 10 — Additional file 9: Supplemental Figure 9. Marker microbial-derived proteins are useful to recognize patients with gastric cancer. A. The abundance of 50 marker proteins in patients with gastric cancer and controls. B. The Euclidean distance between different individuals is shown in the heatmap. C–E. The confusion matrix of the predicted results in the training dataset (C), test dataset (D), and Multi-center cohort (E). [file 40168_2023_1730_MOESM9_ESM.tif]
