# Supplementary material for: In-depth metaproteomics analysis of tongue coating for gastric cancer: a multicenter diagnostic research study
Source: Microbiome. 2024 Jan 8;12:6. doi: 10.1186/s40168-023-01730-8 (PMC10773145; doi:10.1186/s40168-023-01730-8)
Supplement: Supplementary file 12 — Additional file 11: Supplemental File 2. Tongue coating spectral library construction and its characteristics. [file 40168_2023_1730_MOESM11_ESM.docx]

**Characteristics of tongue coating spectral library**

To analyze the DIA data, we built a tongue-coating-specific spectral library. The library includes 16768 proteins and 42023 peptides. Most proteins come from microorganisms (n=15211, 90.71%), and only a small part comes from humans (n=1540, 9.18%) (Supplement Figure 1C). The m/z of peptide precursors in our spectrum library was between 400 Th and 1200 Th, and approximately 85.6% of these precursors were between m/z of 400-850 Th (Supplement Figure 1D). Precursors primarily displayed two (89.7%) or three (9.8%) charges (Supplement Figure 1E). 97.1% of peptides were 7–20 amino acids long, with a median length of 12.4 amino acids, consistent with the properties of trypsinized peptides (Supplement Figure 1F). A total of 4680 proteins were detected with at least three proteotypic peptides, and only 2.9% of proteins were found with more than 10 peptides (Supplement Figure 1G). Additionally, fragments from y ions were more frequently detected than those from b ions due to the collision mode (Supplement Figure 1H).


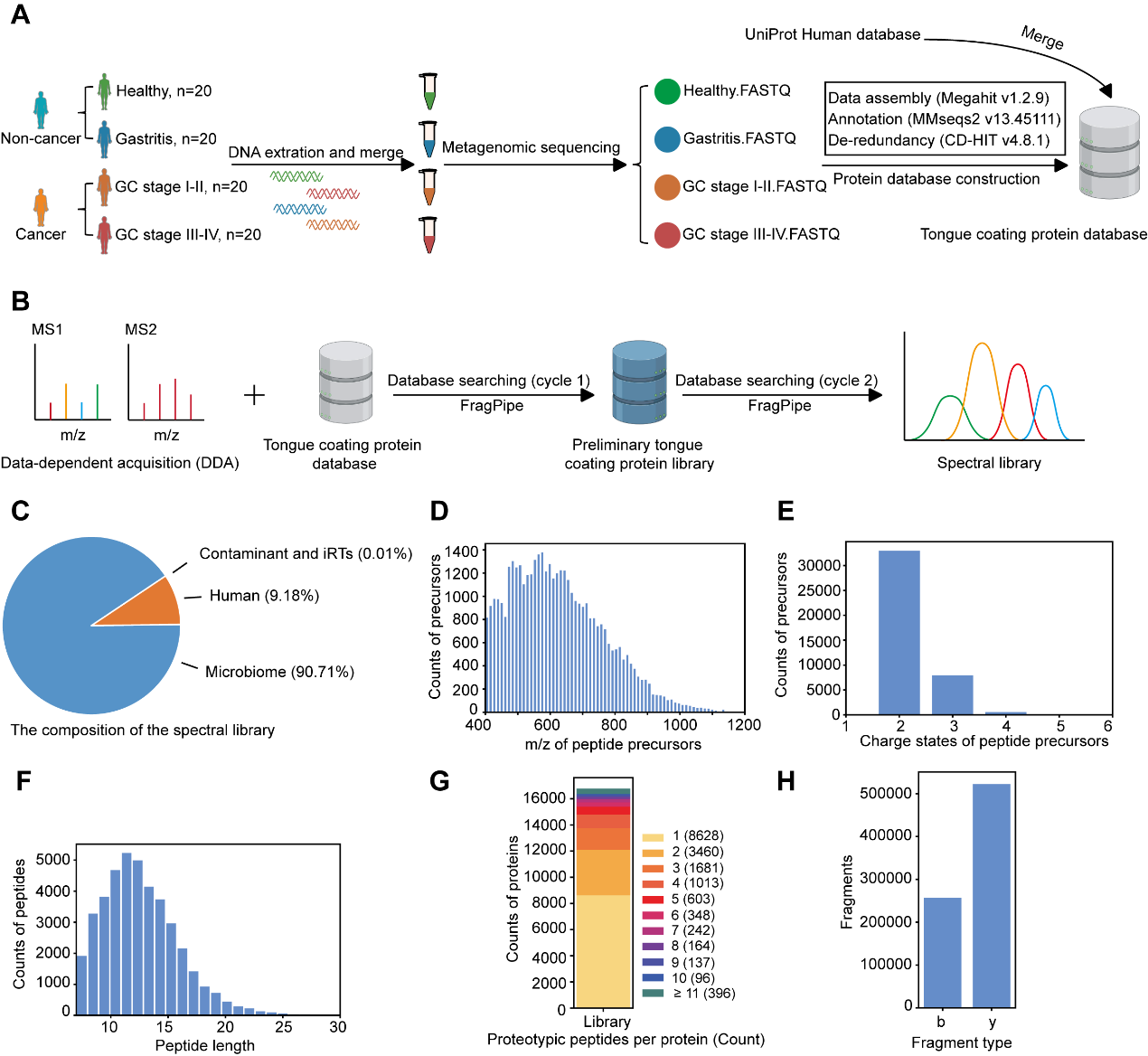


**Supplement Figure 1**

**Tongue coating spectral library construction and its characteristics**

A. Construction workflow of tongue coating specific protein database. B. Construction workflow of tongue coating protein spectral library. C. The type and proportion of entries constituting the tongue coating spectral library. D. Distribution of peptide precursor m/z. E. Counts of different precursor charge states. F. Distribution of identified peptide lengths. G. Numbers of proteotypic peptides for each protein and their corresponding ratios and counts. H. Ion counts of each fragment type.
