# Supplementary material for: In-depth metaproteomics analysis of tongue coating for gastric cancer: a multicenter diagnostic research study
Source: Microbiome. 2024 Jan 8;12:6. doi: 10.1186/s40168-023-01730-8 (PMC10773145; doi:10.1186/s40168-023-01730-8)
Supplement: Supplementary file 13 — Additional file 12: Supplement Table 1. Comparison of clinical information on cancer and non-cancer samples in the ZJC. [file 40168_2023_1730_MOESM12_ESM.docx]

| Total No. |  | ZJC cohort | | | Multi-center cohort | | |
| --- | --- | --- | --- | --- | --- | --- | --- |
|  | Centers | 1 | | | 3 | | |
|  | Samples | 240 | | | 120 | | |
|  |  | Non-cancer  (n=120) | Cancer  (n=120) | *P*-value | Non-cancer  (n=60) | Cancer  (n=00) | *P*-value |
| Age | Median | 65 | 66 |  | 65 | 65 |  |
|  | Range | 46-82 | 46-84 |  | 44-79 | 50-81 |  |
| Gender | Female | 41(34.2%) | 39(32.5%) | 0.784 | 21(35%) | 21(35%) | 1.000 |
|  | Male | 79(65.8%) | 81(67.5%) |  | 39(65%) | 39(65%) |  |
| Smoking | No | 80(66.7%) | 77(64.2%) | 0.684 | 37(61.7%) | 38(63.3%) | 0.850 |
|  | Yes | 40(33.3%) | 43(35.8%) |  | 23(38.3%) | 22(36.7%) |  |
| Alcohol | No | 84(70.0%) | 77(64.2%) | 0.336 | 38(63.3%) | 37(61.7%) | 0.850 |
|  | Yes | 36(30.0%) | 43(35.8%) |  | 22(36.7%) | 23(38.3%) |  |
| Pathology stage | I-II | - | 60(50.0%) |  | - | 29(48.3%) |  |
|  | III-IV | - | 60(50.0%) |  | - | 31(51.7%) |  |

**Supplement Table 1**

Comparison of clinical information on cancer and non-cancer samples in the ZJC cohort and the Multi-center cohort
